# Comprehensive analysis of the clinical significance and molecular mechanism of T-box transcription factor 3 in osteosarcoma

**DOI:** 10.7150/jca.96168

**Published:** 2024-05-30

**Authors:** Yu-Nan Man, Hua Xu, Pei-Jun Chen, Yu Sun, Mao-Lin He

**Affiliations:** 1 Division of Spinal Surgery, The First Affiliated Hospital of Guangxi Medical University, Shuang Yong Road 6, Nanning, Guangxi Zhuang Autonomous Region, P.R. China. 530021.; 2 Center for Education Evaluation & Faculty Development, Guangxi Medical University, Nanning, Guangxi Zhuang Autonomous Region, P.R. China. 530021.; 3 Guangxi Collaborative Innovation Center for Biomedicine, Guangxi Medical University, Nanning, Guangxi Zhuang Autonomous Region, P.R. China. 530021 (Guangxi-ASEAN Collaborative Innovation Center for Major Disease Prevention and Treatment, Guangxi Medical University, Nanning, Guangxi Zhuang Autonomous Region, P.R. China. 530021).

**Keywords:** T-box transcription factor 3 (TBX3), Molecular targets, Osteosarcoma, Metastasis

## Abstract

**Background:** T-box transcription factor 3 (TBX3) has been implicated in various malignant tumors, while its exact involvement in osteosarcoma (OS) remains unknown.

**Methods:** Utilizing microarray data and bulk and single-cell RNA-seq data and qRT-PCR, we compared TBX3 mRNA expression levels in different stages of OS. Diagnostic ability testing and prognosis analysis were conducted to better understand the clinical importance of TBX3. Enrichment analysis was performed using gene groups with biological functions similar to TBX3 in different stages of OS to investigate the potential role of TBX3 in OS progression. In addition, we predicted medications targeted at TBX3 and identified downstream target genes to gain a comprehensive understanding of its therapeutic direction and regulatory mechanism.

**Results:** TBX3 expression was highly upregulated in OS and was predominantly expressed in osteoblastic OS cells, with higher expression levels in metastatic tissues. TBX3 expression appeared somewhat suitable for discriminating between OS and normal samples, as well as different stages of OS. We found that TBX3 increased the malignant development of OS by altering cell cycle and cell adhesion molecules; exisulind and tacrolimus, which are targeted small-molecule medicines, were anticipated to counteract this dysregulation. The expression of CCNA2 could potentially be regulated by TBX3, contributing to OS advancement.

**Conclusion:** TBX3 emerges as a potential biomarker for OS. In-depth research into its underlying molecular processes may offer new perspectives on treating OS.

## 1. Introduction

Osteosarcoma (OS), originating from mesenchymal tissue, is the most prevalent primary malignant bone tumor, constituting approximately 20% of primary malignant bone tumors 1. Primarily affecting the epiphysis of long bones, it carries an average yearly incidence rate of around 4.4 cases per million individuals across all races 2. OS predominantly occurs in teens and is the third deadliest malignancy in this age group 3. Neoadjuvant chemotherapy coupled with limb preservation has become the primary clinical treatment for OS. The combination of immunotherapy, molecular targeted therapy, and traditional Chinese medicine (TCM) has significantly decreased morbidity and mortality among patients with OS. These diverse therapeutic approaches have increased the 5-year survival rate of patients with OS without distant metastases to 60% 4, 5. Despite these advancements, overall survival rates for OS have not improved over the past three decades 6. This can be primarily attributed to the integrity of surgical resection, histological response to treatment, and metastasis and recurrence. Among them, metastasis is the most essential factor contributing to an overall poor survival rate; the five-year survival rate of OS patients with metastasis is <30% 7-9. It is widely acknowledged that OS commonly metastasizes to the lungs and bone tissues, mainly due to the tendency of OS tumor cells to disseminate through the bloodstream, adhere to capillary endothelium, form clots, and undergo proliferation and invasion 10. However, early microscopic lesions are challenging to detect using conventional imaging methods, necessitating the identification of relevant biological markers for the diagnosis of OS, as well as to predict distant metastasis tendency and assess treatment response 11.

T-box transcription factor 3 (TBX3), belonging to the T-box transcription factor protein family, plays a key role in regulating biological development. Previous studies have indicated that Tbx2, as a member of the T-box transcription factor protein family, might be involved in the regulation of cell cycle and cell adhesion. Moreover, Tbx2 has been reported to negatively control Cx43 gene expression, playing a role in regulating osteoblast-like cells and embryo development in rat osteosarcoma cells 12, 13. The structural elements of TBX3 include a DNA-binding T domain, two inhibitory domains, and one activation domain that can function as either inhibitors or agonists of downstream proteins 14. TBX3 has the capability to enhance the activity of phosphoinositide 3-kinase by inhibiting phosphatase and tensin homolog, leading to aberrant cell growth. In addition, TBX3 functions in inhibiting cellular senescence and anti-apoptosis, substantially contributing to aberrant transforming growth factor-β and Wnt/β-catenin signaling pathways 15-19. A direct association reportedly exists between TBX3 expression imbalance and malignant tumor onset and progression. For example, in hepatocellular carcinoma and colorectal cancer, TBX3 expression levels are positively correlated with the grading of cell proliferation markers (Ki-67); further, its high expression is closely related to malignant staging, tumor size, and poor prognosis 20-22. In cervical and gastric cancers, TBX3 enhances the proliferation and invasive ability of tumor cells both *in vivo* and *in vitro* 23, 24. Similarly, TBX3, mediated by transforming growth factor-β1, enhances tumor stemness of bladder cancer cells, accelerating malignant progression 25. Recent investigations into the role of TBX3 in various sarcomas have revealed that TBX3 may be triggered by c-Myc transcription to promote the transformation of human mesenchymal stem cells into sarcomas, leading to enhanced proliferation, migration, and invasion of chondrosarcoma, liposarcoma, and rhabdomyosarcoma. However, TBX3 acts as a tumor suppressor in fibrosarcoma, which is potentially linked to the heterogeneity of distinct sarcoma types 26, 27. A genome-wide analysis involving 17 patients with craniofacial ossifying fibroma, including low-grade OS of the craniofacial region, revealed the association of TBX3 with missense mutations and focal amplification, indicating its pivotal role in the pathogenic pathway 28, 29. Given the preferential occurrence of OS in the epiphyseal end of long bones, a comprehensive analysis with a large sample size is essential to further determine the clinical significance of TBX3 in OS. Therefore, herein we aimed to elucidate the clinical significance of TBX3 in OS by computational biology and meta-analysis based on existing microarray data, high-throughput RNA-seq data, single-cell RNA-seq data, and ChIP-seq data. Moreover, we aimed to investigate the molecular mechanisms and regulatory pathways associated with TBX3, identify potential targeted drugs, and explore its role and functions in OS.

## 2. Materials and methods

### 2.1 Compilation and analysis of pan-cancer data

DepMap, a database for cell function screening, can be utilized to investigate and evaluate potential dependencies of targeted genes in human cancer cell lines; in addition, it allows the retrieval of genomic information from tumor cell lines, assessing their susceptibility to small chemical perturbations 21. This database facilitates the construction of a model of cell population dynamics in CRISPR knockout screens, measured using the Chronos score. A Chronos score of <0 indicates a significant role of the gene in the development of the selected cell line. In other words, decreased expression of the target gene enhances the likelihood of cell death 22. In this study, we downloaded the TBX3 CRISPR (DepMap Public 23Q4+Score Chronos) dataset to evaluate the potential role of TBX3 in the development of diverse malignant tumors. Finally, download expression and prognostic data for pan-cancer samples from the UCSC Xena database, including overall survival (OS), disease-specific survival (DSS), progression-free interval (PFI), and disease-free interval (DFI).

### 2.2 Compilation and analysis of public datasets of OS

#### 2.2.1 Bulk RNA-seq datasets of OS

A comprehensive retrieval of OS-related sequencing and chip data was conducted using the gene expression omnibus (GEO), ArrayExpress, and TARGET databases. This aimed to investigate TBX3 expression levels in OS and control tissues, setting the search term as “(bone tumors OR bone malignancy) OR (osteosarcoma OR osteosarcomas) AND (mRNA OR gene).” and adhering to the following screening criteria: 1. tissues are from OS patients, and 2. TBX3 inclusion in the mRNA expression matrix and 3. dataset comprising OS and control groups, with the expression matrix containing raw data (complete messenger RNA expression data) for standardized processing. Finally, the collected samples should be clearly diagnosed as osteosarcoma or derived from osteosarcoma and have not undergone any radiotherapy or chemotherapy. The exclusion criteria were as follows. 1. non-human samples, 2. inadequate TBX3 expression data in the expression matrix, and 3. lack of a control group in the dataset used to assess TBX3 expression levels. For datasets meeting these filtering requirements, our team performed gene annotation and data format conversion, as previously reported 24. Clinical parameters were then extracted and grouped based on information from public datasets. In this investigation, we sequentially evaluated TBX3 expression in primary OS and normal control (Nc) samples, as well as in metastatic OS and primary OS samples. Statistical assessment of expression differences between groups was achieved using the Wilcoxon rank-sum test. If the group number in the dataset was <3 and the platform was the same, the R package “combat” was employed for data integration after eliminating batch effects 25; otherwise; the dataset was excluded. To further assess the overall expression level of TBX3 in OS, standardized mean difference (SMD) and confidence interval (CI) were calculated by combining sample numbers, expression means, and standard deviations of each group. Finally, publication bias was determined using Egger's and Begg's tests.

#### 2.2.2 Single-cell RNA-seq datasets of OS

One of our objectives was to better understand the functional state and differentiation trajectory of TBX3. For this purpose, we utilized the GSE152048 dataset (GPL24676 Illumina NovaSeq 6000) and adhered to previously published quality control and screening procedures to ensure data reliability 30. The R package “harmony” facilitated data merging, and t-Distributed Stochastic Neighbor Embedding was employed for dimensionality reduction to effectively cluster individual cells 31. To validate annotation accuracy, cells were named using the R package “single R” and referenced to previous literature. Following increased cell annotation and clustering, the R package “monocle2” was utilized to infer and reconstruct the developmental path of TBX3 in OS cells, with additional validation performed using the OS dataset from the TISCH2 database 32.

### 2.3 Cell culture and real-time qPCR

Three human OS cell types (U‐2OS, MG63 and 143B) and human osteoblast cells (hFOB1.19) were obtained from the Cell Bank of the Chinese Academy of Sciences. The human OS cells were cultured in Roswell Park Memorial Institute-1640 medium with 10% fetal bovine serum (Gibco), 100 U/mL penicillin (Gibco) and 100 μg/mL streptomycin (Gibco). Subsequently, total RNA was extracted using TRIzol (Invitrogen, USA). Real-time PCR assay was performed using an ABI Vii7 system (Applied Biosystems, USA), according to the manufacturer's protocol. GAPDH was used as a reference gene. The primer sequences of TBX3 were as follows: forward: 5'-CCCGGTTCCACATTGTAAGAG-3'; reverse: 5'-GTATGCAGTCACAGCGATGAAT-3', the normalization detection method is described above 23.

### 2.4 Clinical significance of TBX3 in patients with OS

To evaluate the accuracy of distinguishing patients with OS based on TBX3 mRNA expression level, receiver operating characteristic (ROC) curves were constructed, and area under the curve (AUC) was calculated using the R package “pROC.” In general, when the AUC value is >0.5 and closer to 1, the results are deemed credible. Summary ROC curves were then derived by integrating the ROC curves of all datasets to comprehensively evaluate the predictive ability of TBX3. Deek's test was used to detect publication bias. Kaplan-Meier curves and univariate Cox regression models were drawn using the R package “survival” to determine the prognostic significance of TBX3 in cancers.

### 2.5 Potential molecular mechanisms of TBX3 in OS

#### 2.5.1 Acquisition of gene groups with similar expression to TBX3 in OS

Gene clusters with high expression similarity are currently considered a robust method for mining target gene regulation and biological functions 33. Accordingly, we first used the limma and limma-voom algorithms to identify differentially expressed genes across all datasets. Upregulated genes, occurring four times or more (frequency of occurrence ≥3 in datasets of metastatic OS samples), were identified after screening for genes with log_2_FC ≥ 1 and *p* < 0.05. Downregulated genes referred to those meeting the frequency of occurrence criteria but displaying log_2_FC ≤ -1 and *p* < 0.05. Genes with expression similar to TBX3 were then identified using the Spearman method; genes with r ≥ 0.3, *p* < 0.05, and frequency of occurrence of ≥4 were screened (frequency of occurrence ≥3 in metastatic OS samples) and defined as genes positively correlated with TBX3 expression. Genes negatively correlated with TBX3 expression were those meeting the frequency of occurrence criteria but displaying r ≤ -0.3 and *p* < 0.05. Genes meeting the screening requirements for both upregulated and positively correlated similar genes were labeled as up-positive genes, with the opposite defined as down-negative genes. These two groups were subsequently subjected to enrichment analysis.

#### 2.5.2 Functional annotation and pathway enrichment analyses

KEGG pathway enrichment analysis was performed using the R package “clusterProfiler” to investigate the potential molecular mechanisms of gene groups with similar expression to TBX3 in OS. Subsequently, based on the R package “GOSemSim” 34, hub genes closely associated with TBX3 were further screened by assessing similarities in molecular functions and cellular localization. The geometric means of biological process and cellular component were employed to measure similarity, identifying molecules with the closest biological function or interaction with TBX3 in OS 35.

### 2.6 Targeted small-molecule therapies for TBX3

Based on the Connectivity Map (CMap) database, we employed the eXtreme Sum (XSum) method to predict potential targeted small-molecule therapies for TBX3. This algorithm (XSum) has demonstrated superior predictive abilities in targeted cancer therapy in comparison to the traditional Kolmogorov-Smirnov method for computing CMap scores 36. For higher precision, the median expression of TBX3 was used to separate high and low expression groups. Differential analysis provided log_2_FC for each group, and the top 100 genes with the largest changes were extracted to obtain the molecular characteristics of TBX3 related in OS. Subsequently, the CMap drug expression matrix, encompassing 1,309 drug expression features following treatment in five cell lines, was utilized to obtain the molecular characteristics of medications. The CMap score, determined using the XSum method, indicated that lower scores were more likely to reverse the molecular characteristics of OS.

### 2.7 Regulatory patterns of TBX3 in OS

To identify downstream genes that might be regulated by TBX3, we employed the KnockTF database, which provides gene expression patterns following targeted transcription factor knockdown 37. In this study, expected target genes were those set to be positively correlated with TBX3 expression and upregulated in both primary OS and metastatic OS samples, with predicted findings from the database. The binding region between them was visualized through screening and downloading ChIP-seq data from Cistrome DB 38.

### 2.8 Statistical analysis

The Wilcoxon rank-sum test and SMD were used to evaluate TBX3 expression level between groups. For SMD, the Cochran's Q-test and I^2^ were utilized to evaluate heterogeneity: if I^2^ > 50% and *p* of chi-square test < 0.05, a random effects model was employed for evaluation; otherwise, a fixed effects model was used. A positive SMD value (i.e., >0) indicated a high degree of expression of the research variable. Statistical significance was considered if the 95% CI of SMD did not contain zero 39. Begg's, Egger's, and Deek's tests were applied to evaluate publication bias, with *p* < 0.05 indicating significant publication bias in SMD results. StataSE-15 (64 bit) was used to plot summary ROC curves and SMD, while R (v4.1.0) was utilized for statistical analyses and visualization. Unless otherwise specified, *p* < 0.05 indicated statistical significance.

## 3. Results

### 3.1 TBX3 expression levels in OS

#### 3.1.1 TBX3 expression levels in bulk RNA-seq datasets of OS

**Figure 1:**
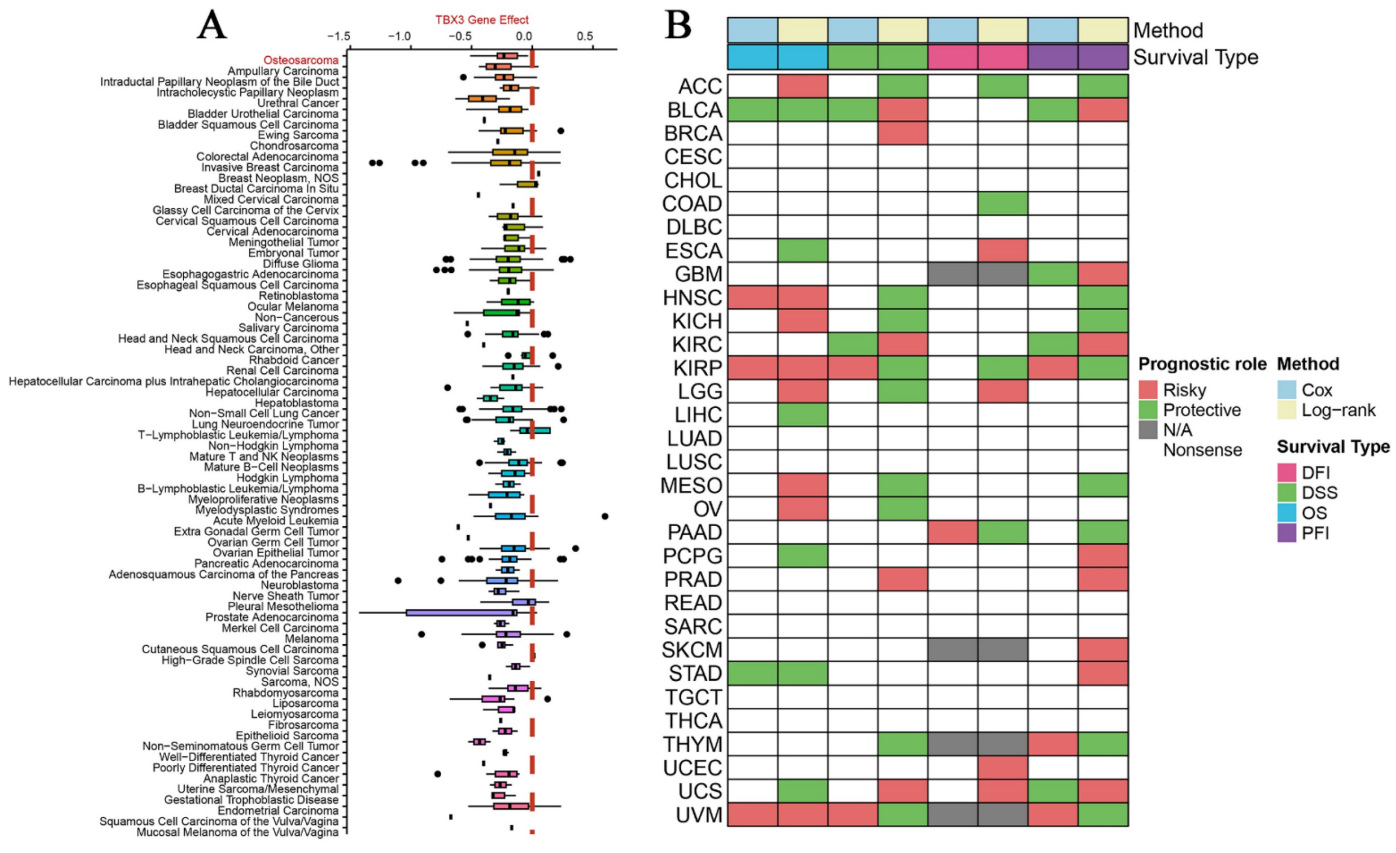
** The vital role and prognostic significance of TBX3 in multiple types of tumors.** (A) Identification of important functions of TBX3 in various malignancies, including OS. (B) Overview of prognostic information of TBX3 in 32 types of tumors. TBX3, T-box transcription factor 3; OS, osteosarcoma.

**Figure 2:**
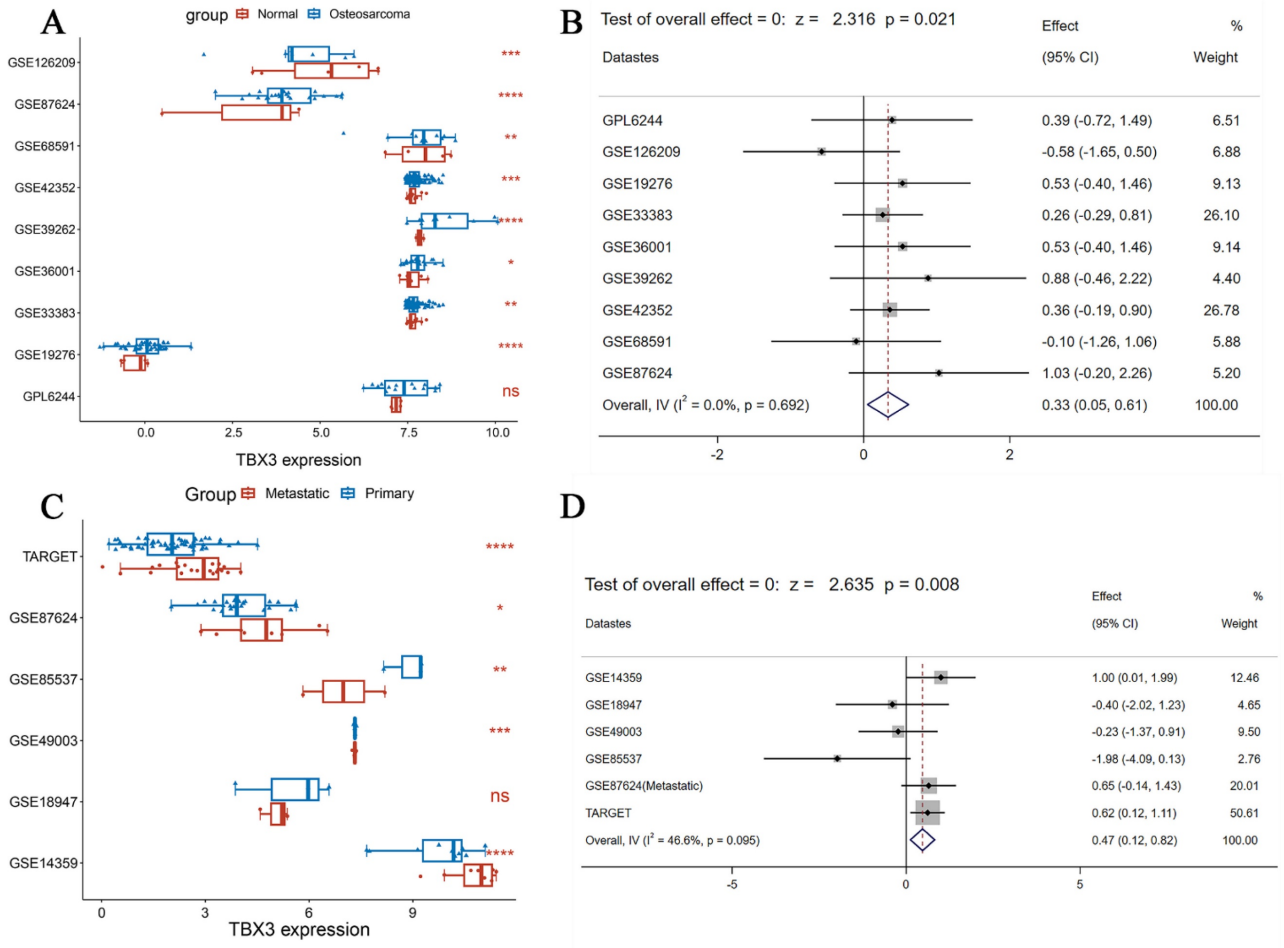
** The integrated investigation of TBX3 mRNA expression level in OS.** (A) The differential expression of TBX3 mRNA between primary OS and normal control samples. (B) Forest diagram of TBX3 mRNA expression in primary OS and normal control samples, prompting for high expression. (C) The differential expression of TBX3 mRNA between primary OS and metastatic OS samples. (D) Forest diagram of TBX3 mRNA expression in metastatic OS and primary OS samples, prompting for high expression. TBX3, T-box transcription factor 3; OS, osteosarcoma. ^ns^*p>* 0.05; *p<0.05; **p <0.01; ***p<0.001; ****p<0.0001.

According to DepMap, the Chronos scores revealed that TBX3 plays an essential function in various malignant tumors, including OS (Figure 1A). Considering the unclear prognostic significance of TBX3 in various cancers, we summarized the correlation between expression of TBX3 with overall survival, disease-specific survival, disease-free interval and progression-free interval based on the univariate Cox regression and Kaplan-Meier models (Figure 1B). Additionally, the forest plot exhibited the prognostic role of TBX3 in cancers by univariate Cox regression method based on four clinical outcomes (Figure S1). To investigate further its expression in the OS datasets, this study encompassed 15 datasets with a total of 517 samples, grouping them based on the information provided by the dataset (primary OS vs. Nc samples; metastatic OS vs. primary OS samples) (Figure S2). Additionally, the detailed clinic parameters along with the TBX3 expression status were showed in Supplementary Table 1. Based on our criteria, the expression matrices of GSE11414 and GSE12865 were merged into GPL6244 (Figure S3A-B). Results from Wilcoxon rank-sum test analysis indicated significantly higher mRNA expression levels of TBX3 in primary OS samples compared to that in Nc samples in GSE19276 (*p* < 0.0001), GSE33383 (*p* < 0.01), GSE36001 (*p* < 0.05), GSE39262 (*p* < 0.0001), GSE42352 (*p* < 0.001), and GSE87624 (*p* < 0.0001). In GSE68591 (*p* < 0.01) and GSE126209 (*p* < 0.001), TBX3 expression was considerably lower in primary OS samples than in Nc samples, while no statistically significant difference in TBX3 expression was observed between the groups in GPL6244 (Figure 2A). In GSE14359 (*p* < 0.0001), GSE87624 (*p* < 0.05), and TARGET (*p* < 0.0001), TBX3 expression in metastatic OS samples was higher than that in primary OS samples. In GSE85537 (*p* < 0.01) and GSE49003 (*p* < 0.001), TBX3 expression in metastatic OS samples was lower, while no statistically significant difference in TBX3 expression was observed between the groups in GSE18947 (Figure 2C). To gain insights into TBX3 expression levels, an integrated study was conducted. TBX3 mRNA expression levels were found to be higher in 317 primary OS samples as compared to those in 52 Nc samples (SMD = 0.33, 95% CI [0.05-0.31], *p* = 0.021) (Figure 2B). Similarly, in comparison to 111 primary OS samples, TBX3 mRNA expression levels were higher in 51 metastatic OS samples (SMD = 0.47, 95% CI [0.12-0.82], *p* = 0.008) (Figure 2D). It is notable that based on heterogeneity results, the fixed effects model was chosen, and publication bias was evaluated for both studies (Figure S3C-D); neither demonstrated any publication bias. Combining the data further revealed that the overall expression level of TBX3 in OS remained significantly higher than in Nc samples (SMD = 0.39, 95% CI [0.17-0.61], *p* = 0.001) (Figure S3E). The entire combined data were analyzed by Begg's and Egger's tests, indicating that the merged results were free of publication bias (Figure S3F-G). Sensitivity analysis findings showed that no dataset contributed to heterogeneity, and the total SMD effect size was statistically significant. Inter-study heterogeneity did not impact the stability of results (Figure S3H).

#### 3.1.2 TBX3 expression level in single-cell RNA-seq datasets and cell lines of OS

**Figure 3:**
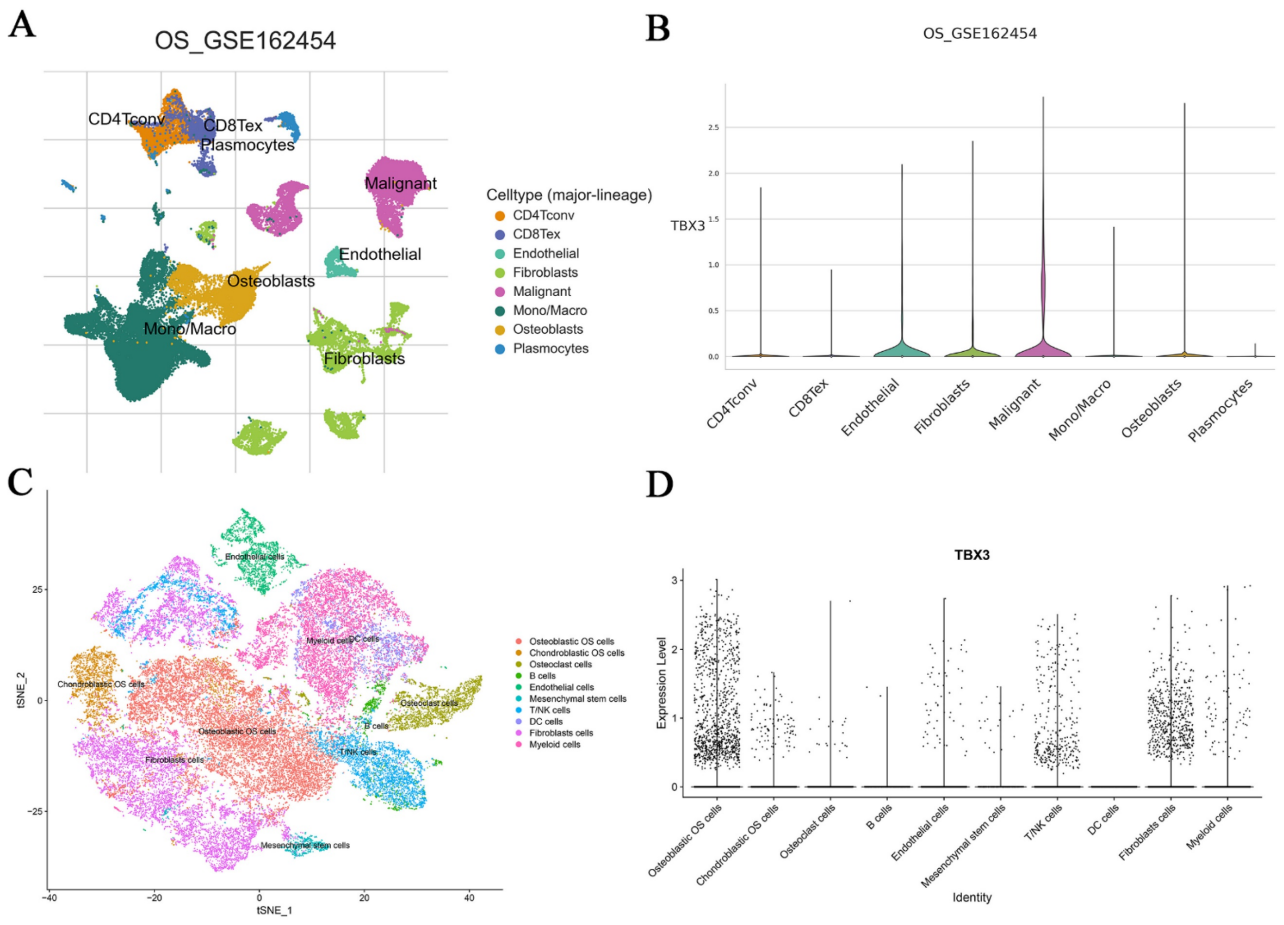
** The mRNA expression levels of TBX3 and its differentiation state was dissected form the perspective of single cells.** (A) Different cell types of OS microenvironment based on the TISH2 database. (B) Expression levels of TBX3 in different types of cells in OS and malignant cells have the highest level of expression among them, followed by osteoblasts cells. (C) Single cells isolated from the OS tissue samples were annotated using singleR and manual. (D) The expression level of TBX3 in overall osteosarcoma samples, mainly enriched in osteoblastic OS cells. TBX3, T-box transcription factor 3; OS, osteosarcoma.

Following the demonstration of elevated TBX3 mRNA expression in bulk RNA-seq datasets of OS, our next objective was to validate this at the single-cell level. According to TISH2, we observed that OS cells exhibited the highest expression level of TBX3, followed by osteoblasts (Figure 3A-B). For a more thorough examination of TBX3 expression in metastatic OS samples, we specifically selected samples from the same tissue source (BC2 Femur-Primary, BC5 Femur-Primary, and BC10 Femur-Metastasis) for subsequent analysis, utilizing GSE152048. After selecting the appropriate latitude for PCA via an elbow plot, we merged samples using the R package “harmony,” and t-Distributed Stochastic Neighbor Embedding was utilized to nonlinear dimensionality reduction. Subsequently, cell subpopulations were named using the R package “singleR” along with manual annotation (Figure 3C, Figure S4A-D). We found that TBX3 was highly expressed in osteoblastic OS cells overall (Figure 3D). Furthermore, in comparison to primary OS cells, TBX3 expression level in metastatic OS cells was higher (Figure S4E). To illustrate, we isolated osteoblastic OS cell subpopulations from metastatic samples and performed single-cell trajectory analysis. With an increase in pseudotime, TBX3 expression in osteoblastic OS cells also tended to increase (Figure S4F-G). Ultimately, we found that compared with human osteoblast cells, TBX3 mRNA level was significantly higher in MG63 and 143B cells lines but not in U-2OS cells, these findings suggest that TBX3 could be a potential clinical biomarker in OS (Figure S5).

### 3.2 Clinical significance of TBX3 overexpression in OS

**Figure 4:**
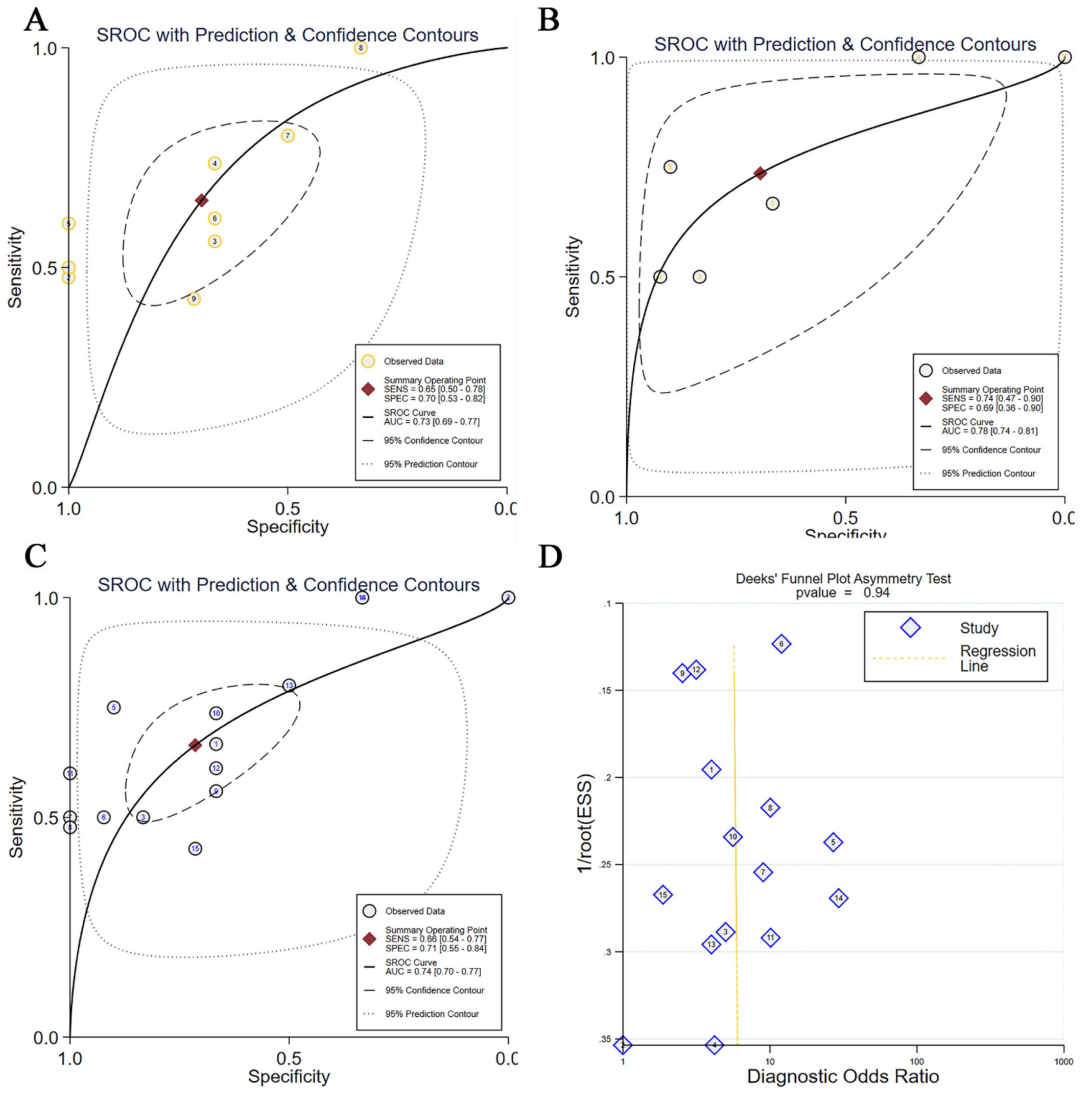
** The discrimination potential of TBX3 in OS.** (A) The sROC with prediction and confidence contours in primary OS and normal control samples, AUC=0.73; (B) The sROC with prediction and confidence contours in metastatic OS and primary OS samples, AUC=0.78; (C) The sROC with prediction and confidence contours in overall OS samples and normal control samples, AUC=0.74; (D) Deek's funnel diagram, which suggested no publication bias (*p* > 0.05). Summary receiver operating characteristic curve; OS, osteosarcoma.

After confirming the elevation of TBX3 expression levels in OS, we aimed to assess the diagnostic potential of TBX3 using primary OS and Nc tissue samples, as well as metastatic OS and primary OS samples. For this purpose, we generated ROC curves (Figure S6A-I, Figure S7A-F) and performed integrated analyses. TBX3 expression showed moderate potential to distinguish between these groups (AUC = 0.73 and 0.78, respectively, Figure 4A-B). Similarly, upon integrating foregoing data and performing comprehensive analyses, TBX3 expression levels were found to satisfactorily discriminate between OS and Nc samples (AUC = 0.74, Figure 4C). Deek's test results revealed no publication bias (Figure 4D). Notably, elevated TBX3 expression was associated with poor prognosis in patients with OS (*p* = 0.01) (Figure S7G).

### 3.3 Potential molecular mechanisms of TBX3 in OS

**Figure 5:**
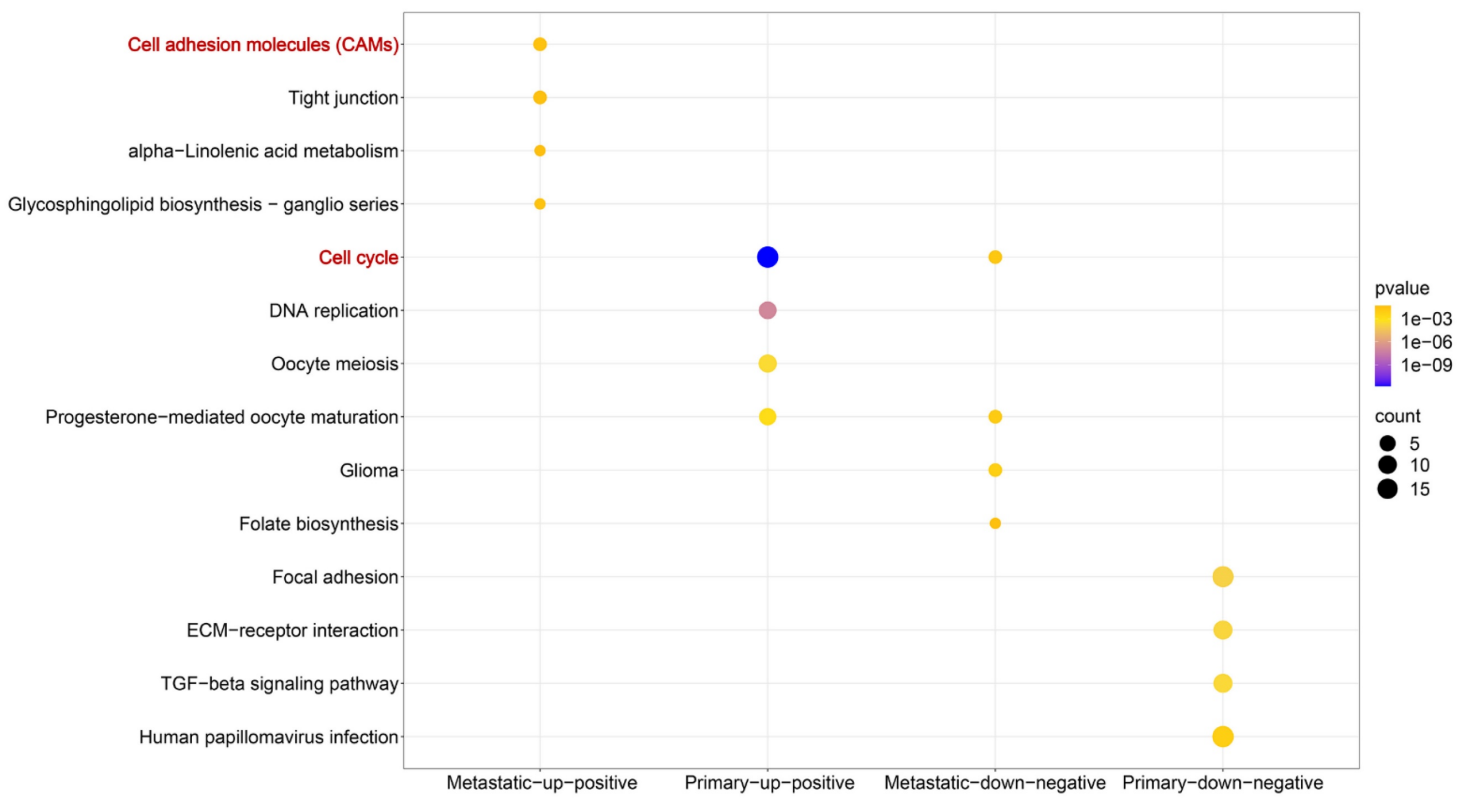
** The potential molecular mechanisms of TBX3 in OS.** Based on metastatic and primary OS samples, we identified four gene groups that are closely linked to TBX3. Wherein, metastatic-up-positive genes were primarily enriched in the Cell Adhesion Molecules (CAMs) pathway while primary-up-positive genes were primarily enriched Cell Cycle pathway. TBX3, T-box transcription factor 3; OS, osteosarcoma.

Building upon the established screening criteria, we identified 285 up-positive and 314 down-negative genes closely linked to TBX3 in primary OS samples; in metastatic OS samples, there were 30 up-positive and 22 down-negative genes (Figure S8A). KEGG pathway enrichment analysis indicated that in metastatic OS samples, up-positive genes were primarily enriched in the cell adhesion molecules pathway, and in primary OS samples, they were enriched in the cell cycle pathway (Figure 5 and Table 1). Subsequently, we focused on metastatic up-positive genes, aiming to uncover genes with biological roles comparable to TBX3 in promoting OS metastasis. The results revealed that PDZD2 was associated with the highest score, indicating a potential interaction that could accelerate malignant development of OS (Figure S8B).

### 3.4 Potential targeted small-molecule therapies for TBX3 in OS

**Figure 6:**
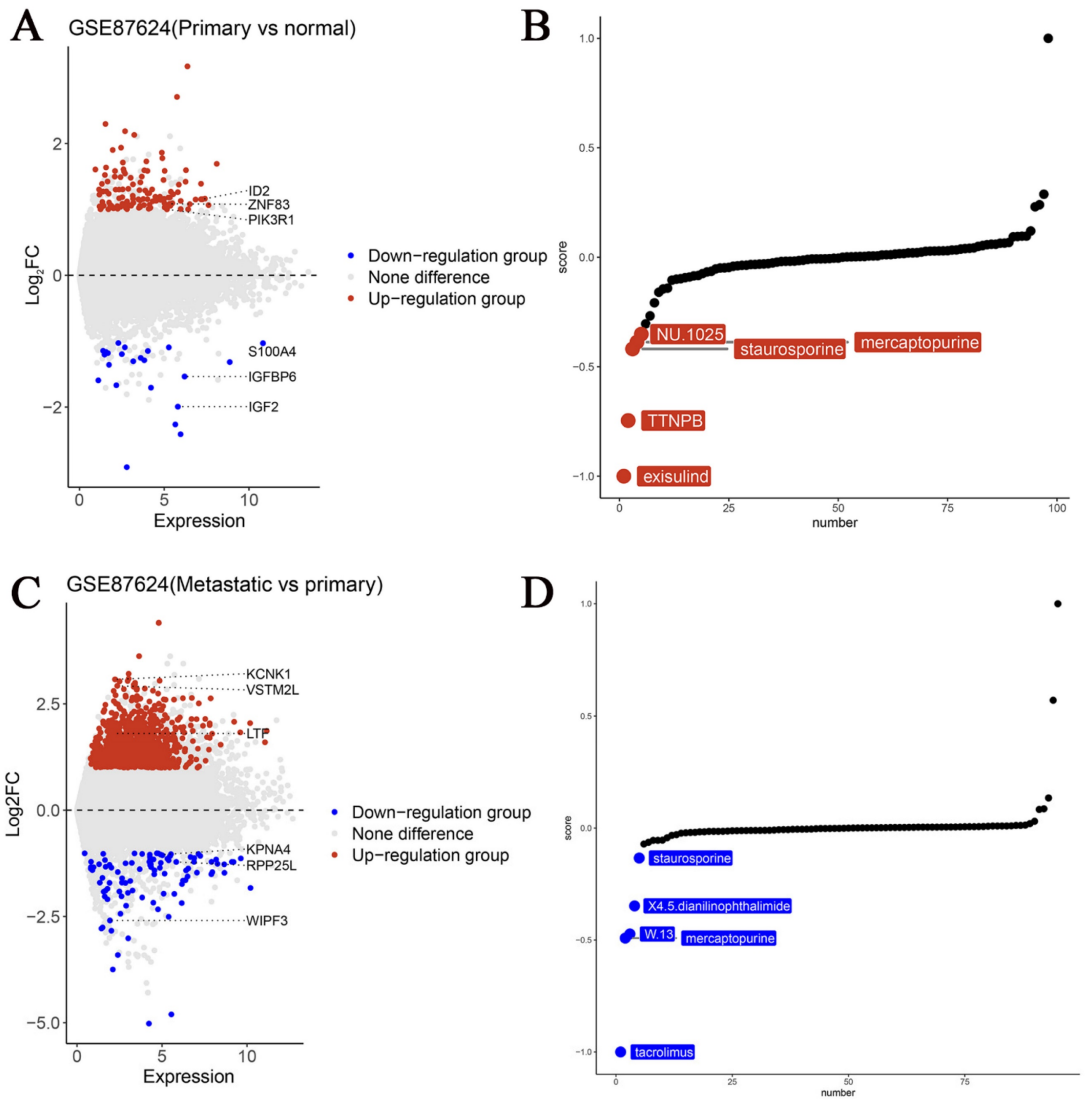
** The potential targeting small molecule medicines of TBX3 in OS.** (A) Genes with differential expression in primary OS and normal control samples were displayed using volcano maps. (B) Top five drugs most likely to target TBX3 in primary OS. (C) Genes with differential expression in metastatic OS and primary OS samples were displayed using volcano maps. (D) Top five drugs most likely to target TBX3 in metastatic OS. TBX3, T-box transcription factor 3; OS, osteosarcoma.

As a potential biomarker for OS, it is critical to identify new targeted medications for precise therapy. Herein we used the GSE87624 dataset, known for its comprehensive sample grouping. Differential analyses were conducted and volcano plots were created based on TBX3 expression levels. Subsequently, using the XSum method and leveraging the “PharmacoGx” R package, we identified potential small molecules targeting TBX3. Notably, exisulind, a small-molecule medication, exhibited outstanding potential for targeted therapy for TBX3 in primary OS samples (Figure 6A-B). We believe that tacrolimus is more likely to target TBX3 in cases with OS metastasis (Figure 6C-D) and the 2D structure and PK/PD parameters of exisulind & tacrolimus are shown in Supplementary Table 2 and Supplementary Table 3 40.

### 3.5 Potential regulatory mechanisms of TBX3 in OS

**Figure 7:**
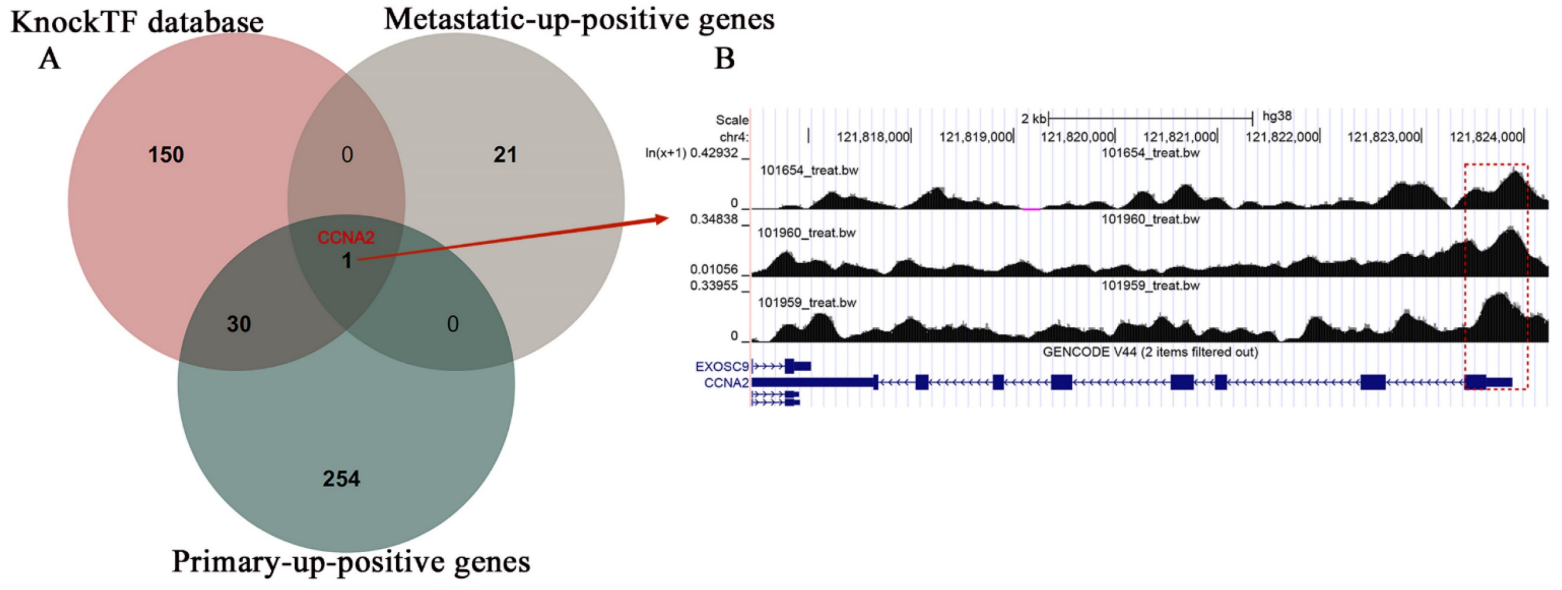
** The potential regulatory mechanisms of TBX3 in osteosarcoma.** (A) Venn diagram showing the potential targeted gene in OS based on the overlap of metastatic-up-positive genes, primary-up-positive genes and KnockTF database. (B) TBX3 could accelerate the expression of CCNA2 and the peaks were marked in the frame. TBX3, T-box transcription factor 3; OS, osteosarcoma.

Utilizing the estimated results from the KnockTF database, we conducted overlapping screening with primary and metastatic up-positive genes, eventually identifying CCNA2 as a potential target gene for TBX3 (Figure 7A). Our result was further validated by Cistrome DB, which displayed a substantial binding peak for TBX3 at the CCNA2 translation start point (Figure 7B).

## 4. Discussion

Previous studies have underscored the challenge in treating OS, a prevalent malignant tumor in children and adolescents. Despite the current standard of limb salvage surgery combined with neoadjuvant chemotherapy maintaining an acceptable overall survival rate for primary OS, the overall survival rate of patients with OS remains stagnant, suggesting a bottleneck in treatment progress 41, 42. The primary cause for this predicament lies in the susceptibility of OS to metastasis. Some individuals exhibit lung metastasis at the time of diagnosis 43. And the overall survival rate of OS patients with metastasis is as low as 20% 44. Considering these challenges, exploring new biological markers to assess OS onset and metastasis becomes imperative for devising novel therapeutic and early preventive strategies.

One promising target is TBX3, which is extensively recognized for its role as an oncogene in various malignancies, including breast cancer, anaplastic thyroid carcinoma and melanoma 45, 46. Notably, TBX3 overexpression has been proven to stimulate melanoma formation and invasion 47. Furthermore, the role of TBX3 in enhancing epithelial-mesenchymal transition across diverse cancers suggests a potential link to tumor dissemination and invasion 48, 49. This seems to be related to its ability to specifically bind to the T-box binding site in the E-cadherin gene promoter, exerting E-cadherin inhibition effects and facilitating self-triggered nuclear translocation 50, 51. Epithelial-mesenchymal transition is recognized to be a critical link in the progression and metastasis of various malignancies, influencing the characteristics and treatment resistance of metastatic tumors. This supports our hypothesis that TBX3 is strongly associated with the incidence and malignant development of OS 52. However, no research has explored the specific function of TBX3 in OS. Thus, we aimed to investigate the clinical importance and mechanism of action of TBX3 in OS using diverse omics data.

First, we confirmed through DepMap that TBX3 plays a critical role in sustaining OS cell proliferation. Based on microarray and high-throughput RNA-seq data, we validated a steady elevation in the mRNA expression level of TBX3 in OS. Besides, TBX3 expression was higher in metastatic OS samples than in primary OS samples. These findings were corroborated using single-cell RNA-seq data, which revealed predominant expression of TBX3 in osteoblastic OS cells. Furthermore, TBX3 mRNA expression levels demonstrated moderate potential to distinguish between primary OS and Nc samples, as well as metastatic OS and primary OS samples. Finally, the association between elevated TBX3 expression and poor prognosis in patients with OS indicated the potential of TBX3 to serve as a biological marker for OS, with focused TBX3 therapy emerging as a viable strategy.

After determining TBX3 expression levels, we sought to investigate its probable mechanism in OS. In primary OS samples, genes positively correlated with TBX3 expression were significantly enriched in the cell cycle pathway, indicating that abnormal TBX3 expression may disrupt cell cycle function in OS. In contrast, in tumor samples with metastasis, the gene group correlated with TBX3 expression was primarily enriched in the cell adhesion molecules pathway. Cell adhesion molecules are macromolecules that mediate the contact and binding between cells or between cells and the extracellular matrix. The shedding of individual tumor cells is the initial stage in tumor invasion and metastasis, a process associated with the loss of adhesion molecules between tumor cells 53. Dysregulation of cell adhesion molecule signal pathway is directly related to the formation, extracellular matrix remodeling, and metastasis of many malignancies, including OS 54-56. Furthermore, PDZD2, proven to be the most similar to TBX3 in terms of cellular localization and biological functions, evidently controls epithelial-mesenchymal transition in OS and enhances the migration of tumor-associated macrophages to promote the malignant biological behavior of OS 57. Thus, our hypothesis posits that TBX3 increases OS development by interfering with the cell cycle pathway. As cancer advances, TBX3 may further control the aberrant behavior of cell adhesion molecules, synergizing with PDZD2 to contribute to the spread of OS. However, further validation is warranted.

A subsequent drug sensitivity study revealed that the majority of small-molecule medicines targeting TBX3 were metabolites and immunosuppressants. Among them, exisulind was identified as having the highest potential among primary OS samples. Functioning as a targeted anti-apoptotic medication, it seems to activate protein kinase G, inducing cell apoptosis 58. In breast cancer, exisulind has been found to cause growth inhibition of tumor cells, leading to G1 phase arrest 59. Considering the potential role of TBX3 in cell cycle disruption in primary OS samples, exisulind may have a certain therapeutic effect. However, investigations into its function in OS are currently lacking. Tacrolimus, a classic immunosuppressive agent, is considered the most beneficial small-molecule medication among metastatic samples. Gao *et al.* employed machine learning to combine and evaluate numerous pharmacological databases, indicating its therapeutic potential in OS 60. Moreover, results from cell dissociation assays suggest that administering tacrolimus, either alone or in combination with other medications, substantially increases cell adhesion in a human keratinocyte cell line (HaCaT cells) and mitigates the loss of cell adhesion induced by human induction. This corresponds to the function of TBX3 in metastatic OS samples, indicating the potential clinical application value of our study 61. Thus, combining the aforementioned medications with existing mainstream clinical chemotherapeutic approaches (MAP) may provide a fresh perspective for enhancing OS treatment. Finally, we anticipate investigating the downstream gene CCNA2, targeted by TBX3. As we previously discovered, CCNA2 has an oncogenic function in upregulating OS expression, demonstrating a similar powerful discriminatory capacity and linkage to poor prognosis 62. Consequently, we propose, for the first time, that TBX3 controls CCNA2 and enhances OS development.

This study has some limitations. First, the small sample sizes in the GEO database are unavoidable, necessitating further multicenter clinical trials and comprehensive prospective investigations for validation. Second, *in vitro* and *in vivo* studies need to be conducted to elucidate the role of TBX3 in OS. Finally, future studies are warranted to validate the synergistic impact of TBX3 and PDZD2, as well as the regulatory influence of TBX3 on CCNA2.

## 5. Conclusions

Considering elevate TBX3 expression in OS, we suggest that TBX3 can serve as a biological marker for OS. TBX3 seems to interact with PDZD2 to regulate CCNA2-mediated dysfunction of cell cycle and cell adhesion factors in OS. Finally, exisulind and tacrolimus offer promising avenues for targeting TBX3 at various stages of OS, presenting prospective treatment options.

## Supplementary Material

Supplementary figures and tables.

## Figures and Tables

**Table 1:** KEGG enrichment analysis based on genes closely linked to TBX3 in OS samples.

ID	Description	*P*-value	Type
hsa04514	Cell adhesion molecules (CAMs)	3.47E-02	Metastatic-up-positive
hsa04530	Tight junction	4.47E-02	Metastatic-up-positive
hsa00592	alpha-Linolenic acid metabolism	4.94E-02	Metastatic-up-positive
hsa00604	Glycosphingolipid biosynthesis - ganglio series	2.99E-02	Metastatic-up-positive
hsa04110	Cell cycle	1.63E-12	Primary-up-positive
hsa03030	DNA replication	1.05E-07	Primary-up-positive
hsa04114	Oocyte meiosis	2.71E-04	Primary-up-positive
hsa04914	Progesterone-mediated oocyte maturation	1.26E-03	Primary-up-positive
hsa05214	Glioma	6.46E-03	Metastatic-down-negative
hsa04914	Progesterone-mediated oocyte maturation	1.08E-02	Metastatic-down-negative
hsa04110	Cell cycle	1.70E-02	Metastatic-down-negative
hsa00790	Folate biosynthesis	4.19E-02	Metastatic-down-negative
hsa04510	Focal adhesion	4.74E-07	Primary-down-negative
hsa04512	ECM-receptor interaction	1.37E-06	Primary-down-negative
hsa04350	TGF-beta signaling pathway	2.66E-06	Primary-down-negative
hsa05165	Human papillomavirus infection	1.13E-04	Primary-down-negative

TBX3, T-box transcription factor 3; OS, osteosarcoma.
