# Supplementary material for: Comprehensive analysis of the clinical significance and molecular mechanism of T-box transcription factor 3 in osteosarcoma
Source: J Cancer. 2024 May 30;15(12):4007–19. doi: 10.7150/jca.96168 (PMC11190752; doi:10.7150/jca.96168)
Supplement: Supplementary file 1 — Supplementary figures and tables. [file jcav15p4007s1.pdf]

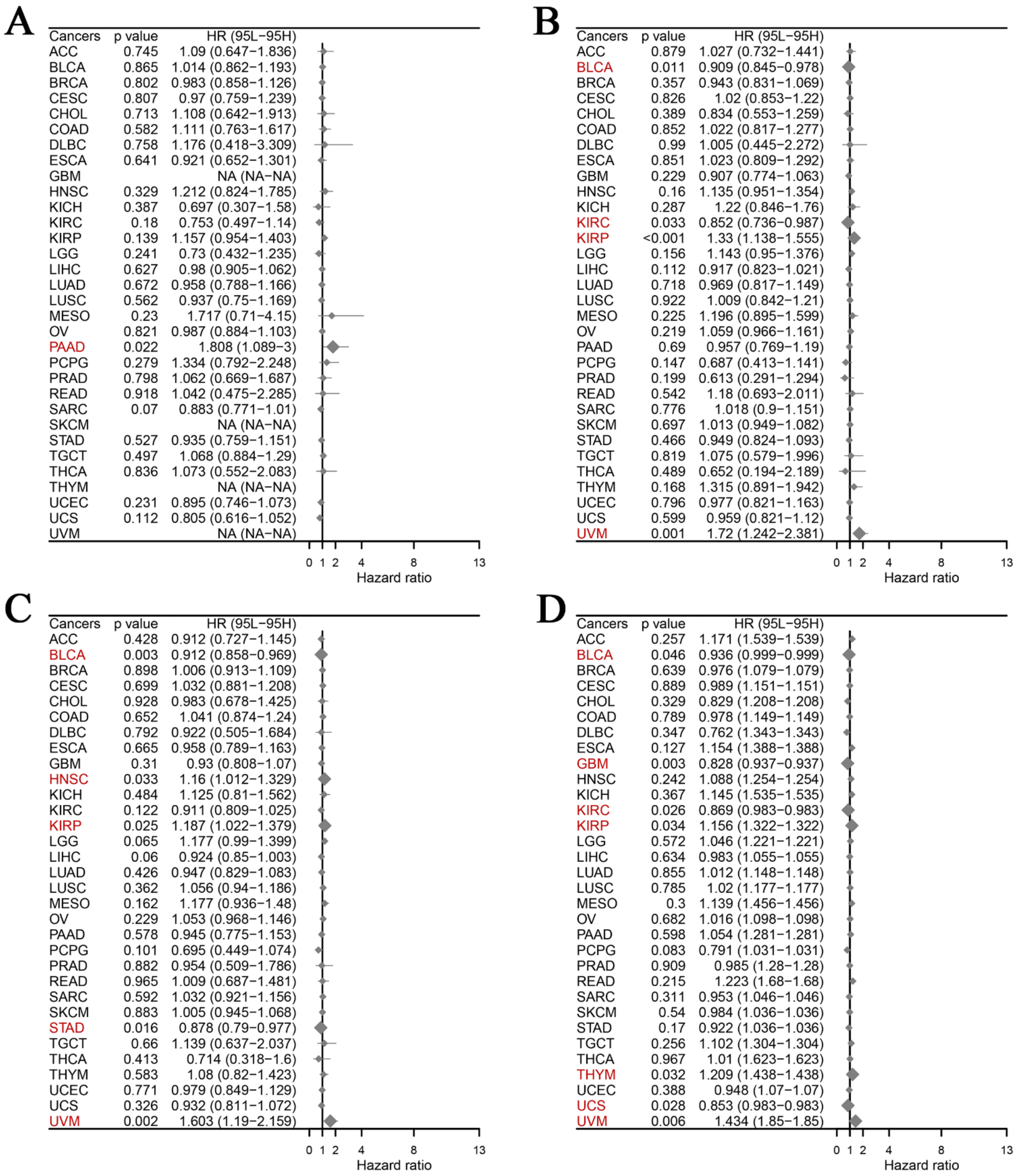

Additional Fig. S1. (A) Relation of TBX3 expression with disease-free interval of 32 types tumors patients. (B) Relation of TBX3 expression with disease-specific survival of 32 types tumors patients. (C) Relation of TBX3 expression with overall survival of 32 types tumors patients. (D) Relation of TBX3 expression with progression-free interval of 32 types tumors patients.

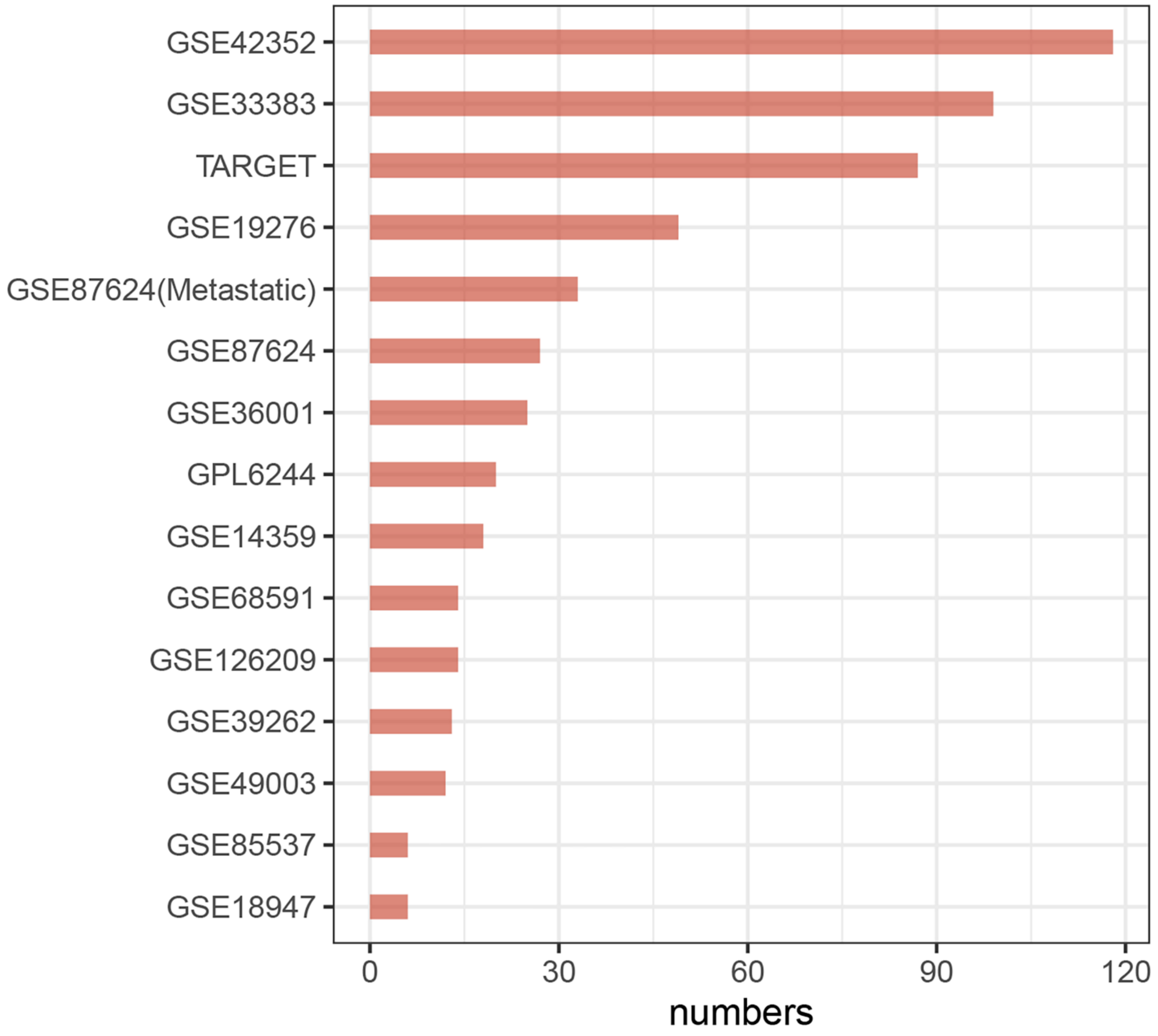

Additional Fig. S2. Datasets included in the study and their sample numbers.

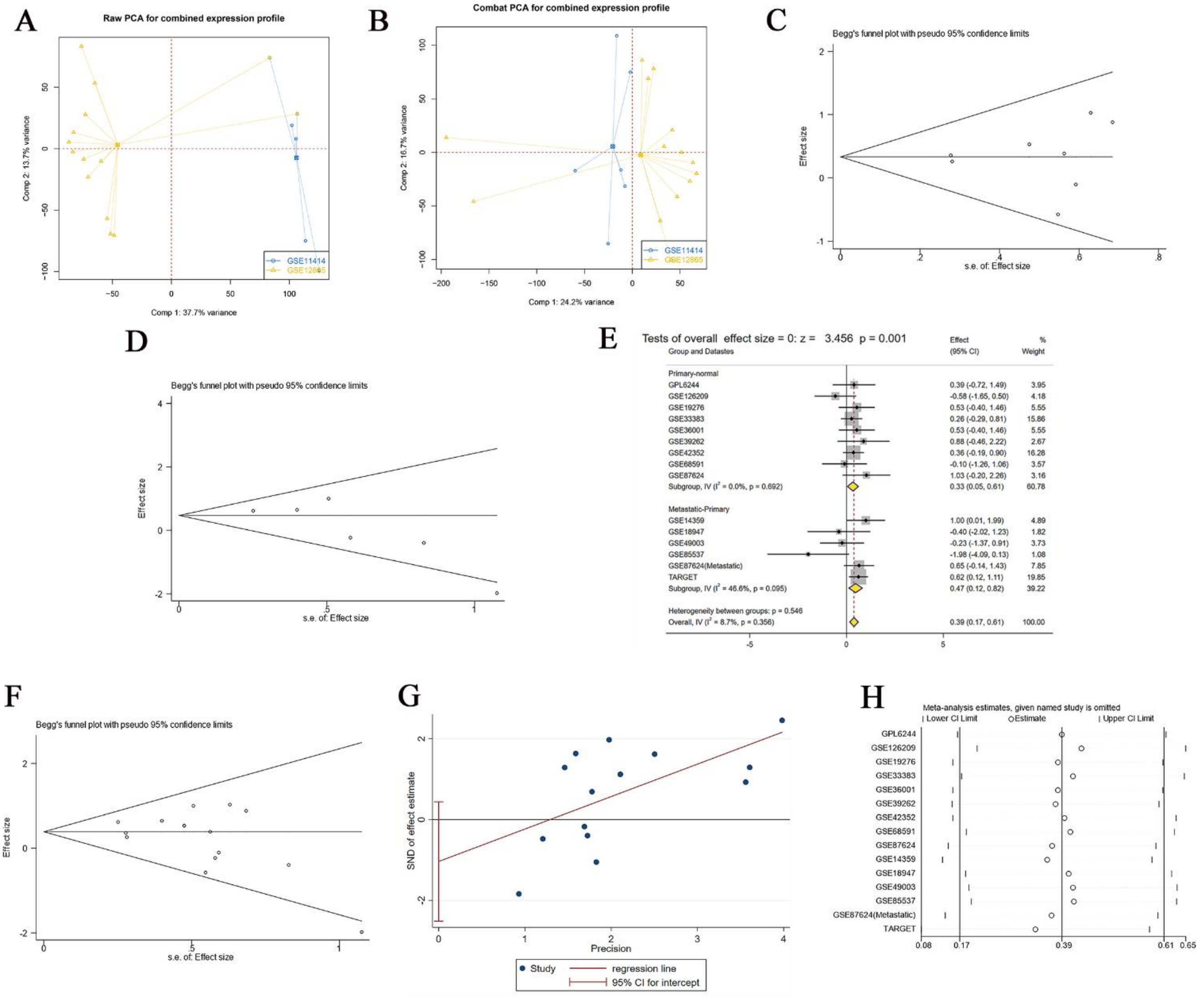

Additional Fig. S3. (A) PCA dimensionality reduction before datasets merging (GSE11414 and GSE12865). (B) PCA dimensionality reduction after datasets merging (GSE11414 and GSE12865). (C) Begg's test showing no publication bias in the analysis of integrating primary OS samples (*p*=0.602). (D) Begg's test showing no publication bias in the analysis of integrating metastatic OS samples (*p*=0.133). (E) Forest diagram of TBX3 mRNA expression in overall OS samples and normal control samples, prompting for high expression. (F) Begg's test showing no publication bias in the analysis of integrating overall OS samples (*p*=0.198). (G) Egger's test showing no publication bias in the analysis of integrating overall OS samples (*p*=0.153). (H) Sensitivity analysis of integration results.

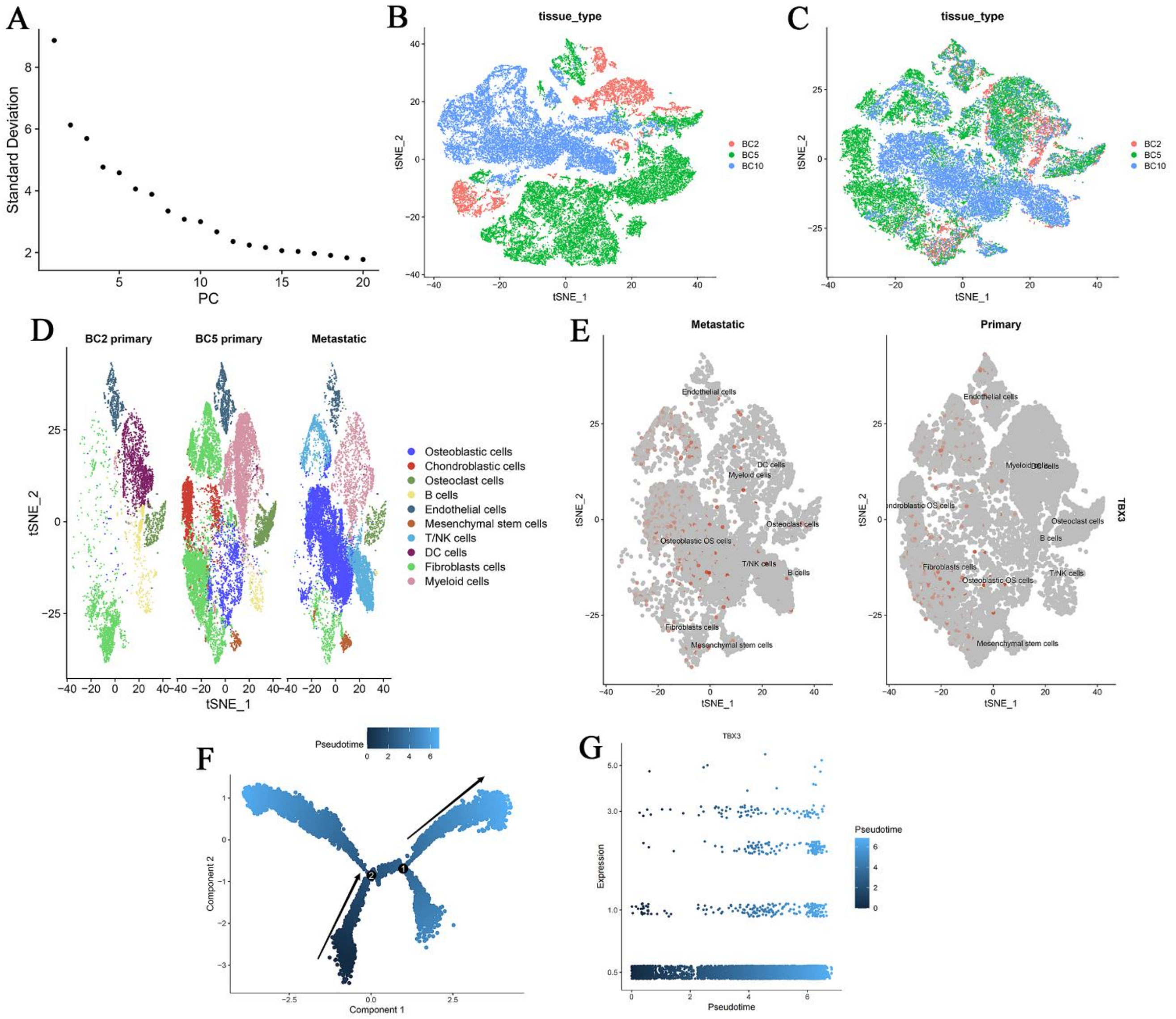

Additional Fig. S4. (A) Elbow plot for latitude selection. (B) Different cell types of OS microenvironment before using R package “harmony” to merge samples. (C) Different cell types of OS microenvironment after using R package “harmony” to merge samples. (D). Different cell types of OS microenvironment based on the different tissue. (E) TBX3’s expression is more elevated in osteoblastic osteosarcoma cells in metastatic samples compared to primary samples. (F) Construction of cell trajectories of TBX3 in osteoblastic OS cells subpopulations based on metastatic samples. (G) As the pseudotime extended, the expression level of TBX3 showed an increasing tendency. TBX3, T-box transcription factor 3; OS, osteosarcoma.

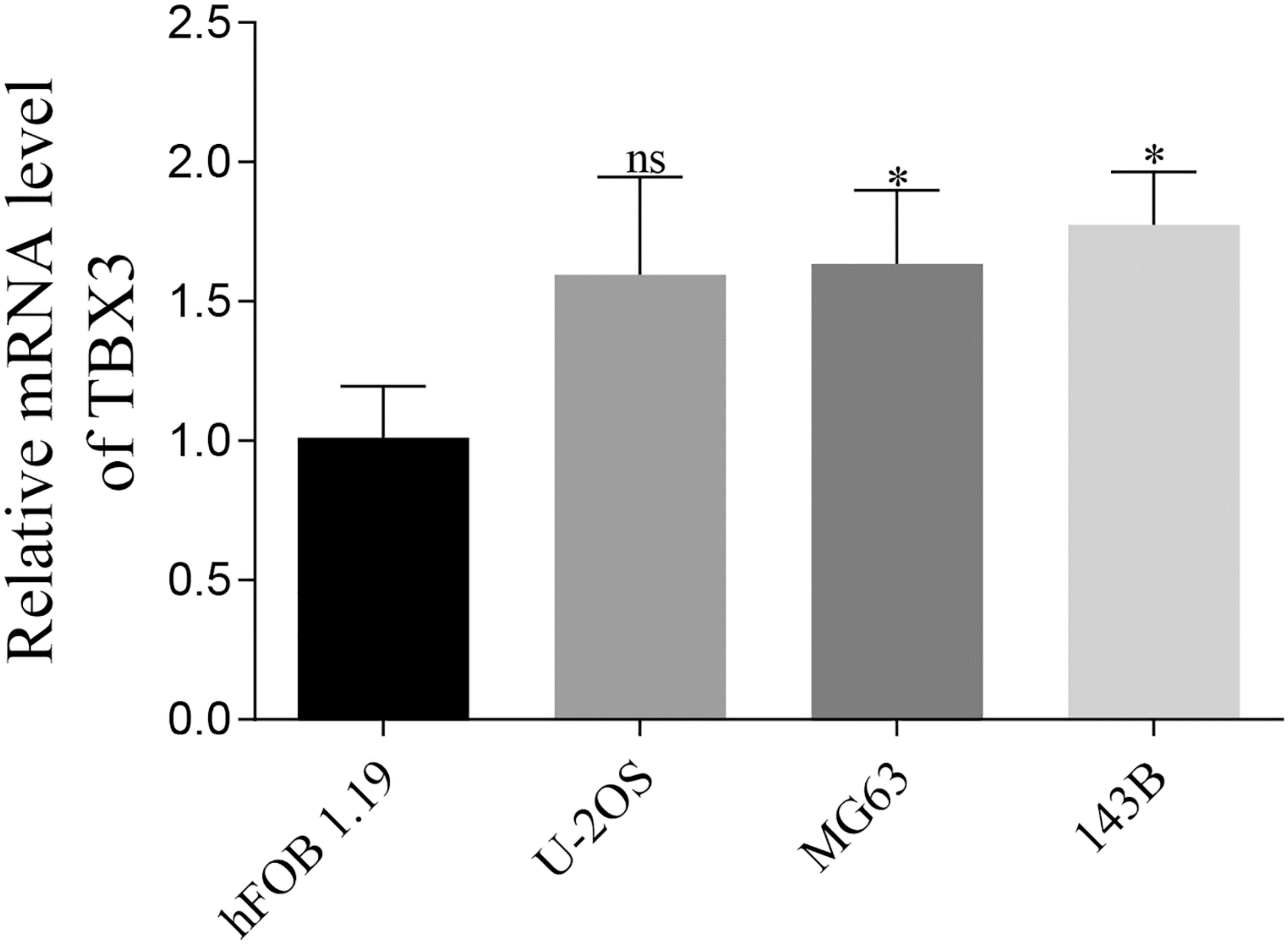

Additional Fig. S5. TBX3 mRNA expression in human OS cell lines (MG63 and 143B) was significantly higher than that in human osteoblast cells (hFOB1.19), but not significantly in U-2OS cells.

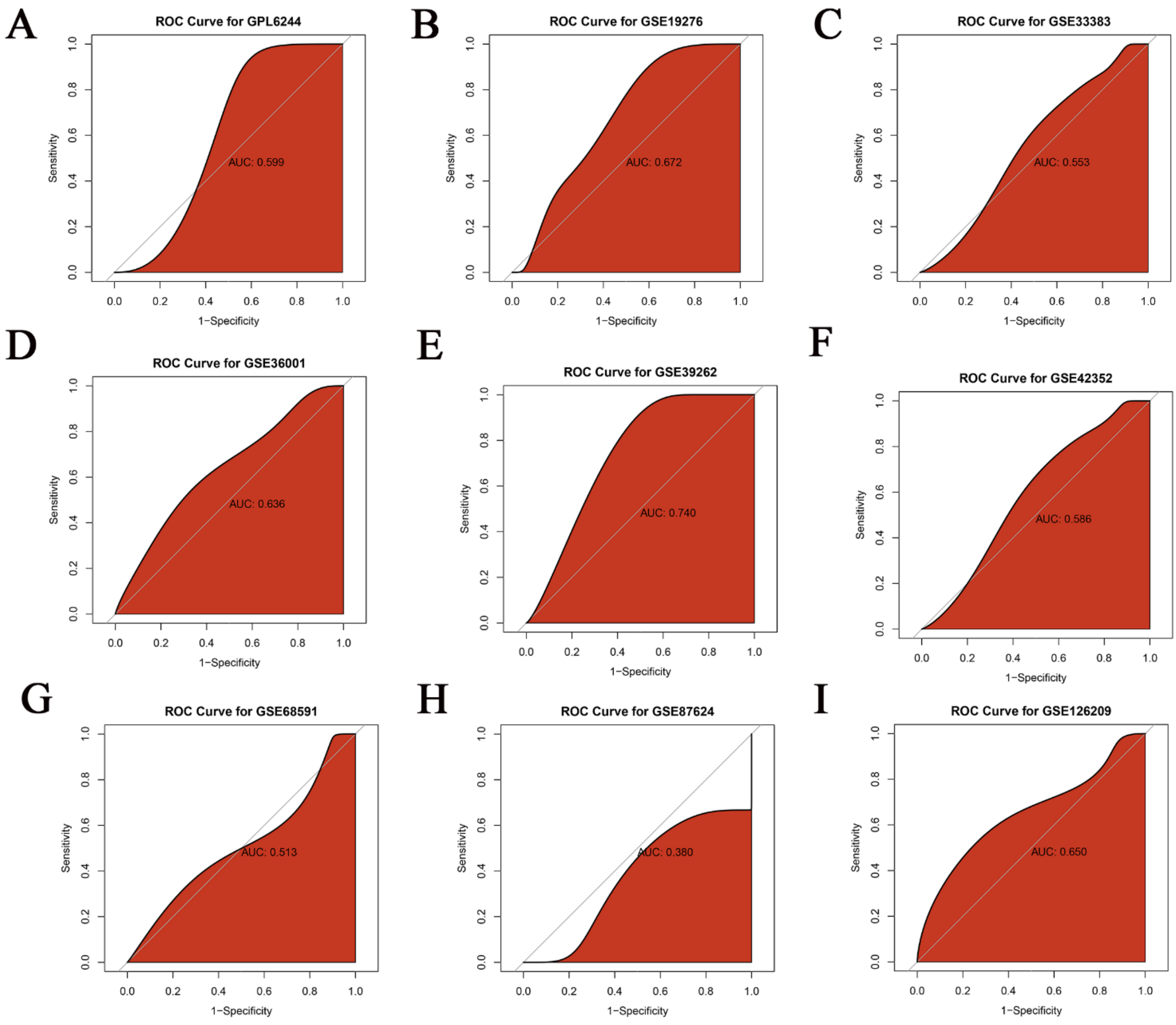

Additional Fig. S6. ROC curves of TBX3 in GEO datasets of primary OS samples.

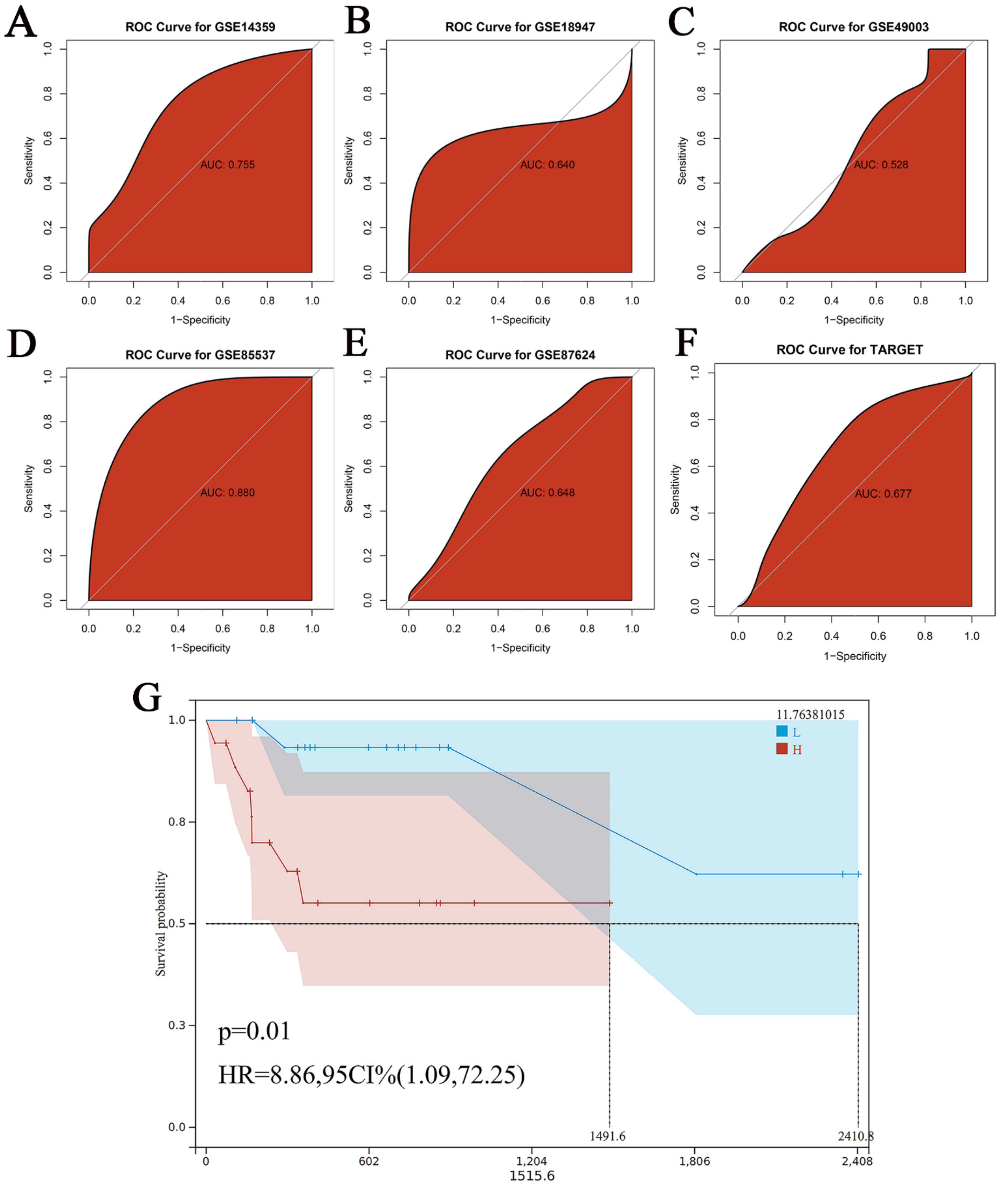

Additional Fig. S7. (A-F) ROC curves of TBX3 in GEO datasets of metastatic OS samples. (G) TBX3 high expression was related to shorter survival time of OS patients.

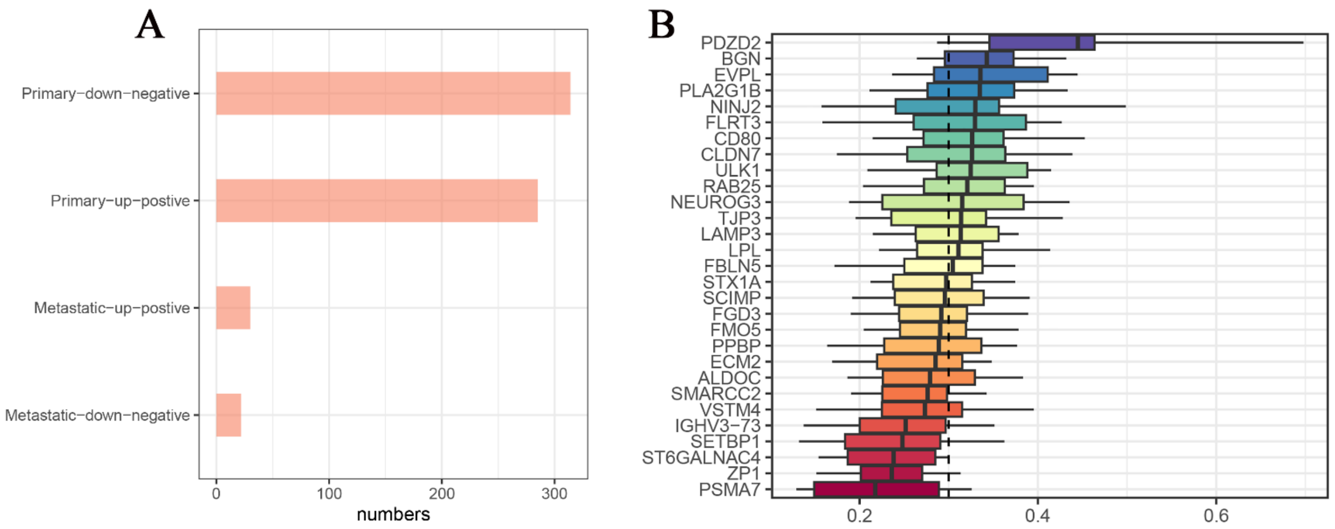

Additional Fig. S8. (A) Genes closely linked to TBX3 in primary and metastatic OS samples. (B) GO friend analysis revealed that PDZD2 got the highest score.

Supplementary table1. The detailed clinic parameters along with the TBX3 expression status in 517 samples

Samples	TBX3 expression status	Platform	Source
GSM288005	7.2392	GPL6244	normal human osteoblasts
GSM288006	7.25706	GPL6244	normal human osteoblasts
GSM322691	6.64905	GPL6244	osteosarcoma tumor 177
GSM322692	6.79037	GPL6244	osteosarcoma tumor 177
GSM322693	6.08054	GPL6244	osteosarcoma tumor 178
GSM322694	6.35785	GPL6244	osteosarcoma tumor 178
GSM322695	8.34348	GPL6244	osteosarcoma tumor 179
GSM322696	8.35211	GPL6244	osteosarcoma tumor 179
GSM322697	7.60309	GPL6244	osteosarcoma tumor 180
GSM322698	7.68539	GPL6244	osteosarcoma tumor 180
GSM322699	6.53589	GPL6244	osteosarcoma tumor 182
GSM322700	6.8786	GPL6244	osteosarcoma tumor 182
GSM322701	8.33629	GPL6244	osteosarcoma tumor 183
GSM322702	8.48162	GPL6244	osteosarcoma tumor 183
GSM288005	7.2392	GPL6244	normal human osteoblasts
GSM288006	7.25706	GPL6244	normal human osteoblasts
GSM288007	7.32452	GPL6244	OS cell line 1
GSM288008	7.34544	GPL6244	OS cell line 1
GSM288009	7.92875	GPL6244	OS cell line 2
GSM288010	7.95555	GPL6244	OS cell line 2
GSM359139	11.15462827	GPL96	conventional osteosarcoma tissue, female, 15 years, femur, grade 3
GSM359140	11.48824184	GPL96	conventional osteosarcoma tissue, female, 15 years, femur, grade 3
GSM359141	10.72766524	GPL96	osteosarcoma lung metastasis tissue, female, 45 years, lung, grade 1
GSM359142	9.844705764	GPL96	osteosarcoma lung metastasis tissue, female, 45 years, lung, grade 1
GSM359143	10.65731845	GPL96	conventional osteosarcoma tissue, female, 74 years, femur, grade 2
GSM359144	10.35887094	GPL96	conventional osteosarcoma tissue, female, 74 years, femur, grade 2
GSM359145	11.20756347	GPL96	osteosarcoma lung metastasis tissue, female, 37 years, lung, grade 2
GSM359146	11.08061769	GPL96	osteosarcoma lung metastasis tissue, female, 37 years, lung, grade 2
GSM359147	10.06622376	GPL96	conventional osteosarcoma tissue, male, 7 years, femur, grade 2
GSM359148	10.60269887	GPL96	conventional osteosarcoma tissue, male, 7 years, femur, grade 2
GSM359149	10.58327085	GPL96	conventional osteosarcoma tissue, male, 17 years, femur, grade 3
GSM359150	9.8166637	GPL96	conventional osteosarcoma tissue, male, 17 years, femur, grade 3
GSM359151	10.70649602	GPL96	osteosarcoma lung metastasis tissue, male, 40 years, lung, grade 3
GSM359152	10.66790986	GPL96	osteosarcoma lung metastasis tissue, male, 40 years, lung, grade 3
GSM359153	11.01164658	GPL96	osteosarcoma lung metastasis tissue, female, 21 years, lung, grade 3
GSM359154	10.79701298	GPL96	osteosarcoma lung metastasis tissue, female, 21 years, lung, grade 3
GSM359155	10.325193	GPL96	conventional osteosarcoma tissue, male, 23 years, tibia, grade 3
GSM359156	10.89504506	GPL96	conventional osteosarcoma tissue, male, 23 years, tibia, grade 3
GSM469260	6.575917361	GPL570	low metastatic potential cell subline of Sosp-9607, biological rep1
GSM469261	5.970393538	GPL570	low metastatic potential cell subline of Sosp-9607, biological rep2
GSM469262	5.371558863	GPL570	high metastatic potential cell subline of Sosp-9607, biological rep1
GSM469263	5.213347282	GPL570	high metastatic potential cell subline of Sosp-9607, biological rep2
GSM469264	3.867896464	GPL570	low metastatic potential cell subline of Saos-2

GSM469265	4.584962501	GPL570	high metastatic potential cell subline of Saos-2
GSM481071	0.659870635	GPL6848	Femaleleft tibia 76 years
GSM481072	-0.075334075	GPL6848	Femaleleft tibia 76 years
GSM481073	0.131947515	GPL6848	Maleleft pelvis 39 years
GSM481074	0.53060961	GPL6848	Maleleft pelvis 39 years
GSM481075	0.372122285	GPL6848	Femaleright femur19 years
GSM481076	0.051123855	GPL6848	Femaleright femur19 years
GSM481077	0.065560815	GPL6848	Male right femur14 years
GSM481078	-0.143960713	GPL6848	Male right femur14 years
GSM481079	0.18720674	GPL6848	Male left femur12 years
GSM481080	-0.15529299	GPL6848	Male left femur12 years
GSM481081	-0.08783675	GPL6848	Female right humerus15 years
GSM481082	0.157060145	GPL6848	Female right humerus15 years
GSM481083	-0.23142765	GPL6848	Female right humerus27 years
GSM481084	-0.46128845	GPL6848	Female right tibia37 years
GSM481085	-0.291349425	GPL6848	Female right tibia37 years
GSM481086	-0.610448835	GPL6848	Female right femur13 years
GSM481087	-0.8345151	GPL6848	Female right femur13 years
GSM481088	0.438986315	GPL6848	Female tibia7 years
GSM481089	-0.111478335	GPL6848	Female tibia7 years
GSM481090	-0.68950318	GPL6848	Female sacrum 29 years
GSM481091	-0.531173235	GPL6848	Female sacrum 29 years
GSM481092	0.062672614	GPL6848	Male right tibia15 years
GSM481093	1.3186114	GPL6848	Male right tibia15 years
GSM481094	0.283712389	GPL6848	Female right femur 15 years
GSM481095	0.533736705	GPL6848	Female right femur 15 years
GSM481096	0.5779624	GPL6848	Male left tibia 18 years
GSM481097	0.182408335	GPL6848	Male left tibia 18 years
GSM481098	0.4605074	GPL6848	Male right femur 18 years
GSM481099	0.61184002	GPL6848	Male right femur 18 years
GSM481100	0.546012865	GPL6848	Maleleft tibia17 years
GSM481101	0.161092515	GPL6848	Maleleft tibia17 years
GSM481102	-1.1785841	GPL6848	Female left femur 14 years
GSM481103	-0.77316331	GPL6848	Female left femur 14 years
GSM481104	0.46350908	GPL6848	Female left femur 24 years
GSM481105	0.43017245	GPL6848	Female left femur 24 years
GSM481106	-0.233511449	GPL6848	Male left tibia 17 years
GSM481107	-0.1928854	GPL6848	Male right pelvis 67 years
GSM481108	0.288132658	GPL6848	Male right pelvis 67 years
GSM481109	-0.006337165	GPL6848	Male right 8th rib 18 years
GSM481110	-0.261027095	GPL6848	Male right 8th rib 18 years
GSM481111	0.26095676	GPL6848	Male left femur 15 years
GSM481112	0.33114934	GPL6848	Male left femur 15 years
GSM481113	-1.283824915	GPL6848	Male left calcaneum 16 years
GSM481114	-0.55947781	GPL6848	Male left calcaneum 16 years
GSM481115	-0.120723725	GPL6848	Female 49 years

GSM481116	0.08402205	GPL6848	Female femur head 75 years
GSM481117	-0.592035317	GPL6848	Male 71 years
GSM481118	0.01032257	GPL6848	Male 56 years
GSM481119	-0.67908096	GPL6848	Male 79 years
GSM717846	7.725149434	GPL10295	220-Msc
GSM717847	7.581945571	GPL10295	240-Msc
GSM717848	7.70788472	GPL10295	Kaat-Msc
GSM717849	7.582494941	GPL10295	MSC_001_OS
GSM717850	7.610370658	GPL10295	MSC_002_OS
GSM717851	7.499490509	GPL10295	MSC_003_OS_2
GSM717852	7.475565305	GPL10295	MSC_006_OS
GSM717853	7.557016603	GPL10295	MSC_009_OS
GSM717854	7.56583806	GPL10295	MSC_HD3
GSM717855	7.643666737	GPL10295	MSC_HD5
GSM717856	7.581932934	GPL10295	MSC_MH
GSM717857	7.569726137	GPL10295	MSC_TD_001
GSM825623	8.035499018	GPL10295	Osteoblast 220-OB
GSM825624	7.895234483	GPL10295	Osteoblast 240-OB
GSM825625	7.754401898	GPL10295	Osteoblast Kaat-OB
GSM825626	7.983967697	GPL10295	High-grade osteosarcoma pre-chemotherapy biopsy L428int
GSM825627	7.704090315	GPL10295	High-grade osteosarcoma pre-chemotherapy biopsy L432int
GSM825628	7.51312935	GPL10295	High-grade osteosarcoma pre-chemotherapy biopsy L436int
GSM825629	8.107275821	GPL10295	High-grade osteosarcoma pre-chemotherapy biopsy L975int
GSM825630	7.670882398	GPL10295	High-grade osteosarcoma pre-chemotherapy biopsy L997int
GSM825631	7.553985172	GPL10295	High-grade osteosarcoma pre-chemotherapy biopsy L1016int
GSM825632	7.574400577	GPL10295	High-grade osteosarcoma pre-chemotherapy biopsy L1085int
GSM825633	7.661105847	GPL10295	High-grade osteosarcoma pre-chemotherapy biopsy L1368int
GSM825634	7.688257221	GPL10295	High-grade osteosarcoma pre-chemotherapy biopsy L1369int
GSM825635	7.496317287	GPL10295	High-grade osteosarcoma pre-chemotherapy biopsy L1370int
GSM825636	7.673973769	GPL10295	High-grade osteosarcoma pre-chemotherapy biopsy L1372int
GSM825637	7.797207625	GPL10295	High-grade osteosarcoma pre-chemotherapy biopsy L1376int
GSM825638	7.770380208	GPL10295	High-grade osteosarcoma pre-chemotherapy biopsy L1378int
GSM825639	7.640796799	GPL10295	High-grade osteosarcoma pre-chemotherapy biopsy L1382int
GSM825640	7.543910411	GPL10295	High-grade osteosarcoma pre-chemotherapy biopsy L1385int
GSM825641	7.821689727	GPL10295	High-grade osteosarcoma pre-chemotherapy biopsy L1386int
GSM825642	7.636564422	GPL10295	High-grade osteosarcoma pre-chemotherapy biopsy L2068int
GSM825643	7.727378325	GPL10295	High-grade osteosarcoma pre-chemotherapy biopsy L2178int
GSM825644	7.728246934	GPL10295	High-grade osteosarcoma pre-chemotherapy biopsy L2292int
GSM825645	7.444334435	GPL10295	High-grade osteosarcoma pre-chemotherapy biopsy L2295int
GSM825646	7.452363696	GPL10295	High-grade osteosarcoma pre-chemotherapy biopsy L2296int
GSM825647	7.776465692	GPL10295	High-grade osteosarcoma pre-chemotherapy biopsy L2297int
GSM825648	7.497378261	GPL10295	High-grade osteosarcoma pre-chemotherapy biopsy L2301int
GSM825649	7.646537053	GPL10295	High-grade osteosarcoma pre-chemotherapy biopsy L2302int
GSM825650	7.435562819	GPL10295	High-grade osteosarcoma pre-chemotherapy biopsy L2347int
GSM825651	7.709310064	GPL10295	High-grade osteosarcoma pre-chemotherapy biopsy L2376int
GSM825652	7.481747729	GPL10295	High-grade osteosarcoma pre-chemotherapy biopsy L2611int

GSM825653	7.749049819	GPL10295	High-grade osteosarcoma pre-chemotherapy biopsy L2613int
GSM825654	7.436044038	GPL10295	High-grade osteosarcoma pre-chemotherapy biopsy L2614int
GSM825655	7.51551564	GPL10295	High-grade osteosarcoma pre-chemotherapy biopsy L2615int
GSM825656	7.474996	GPL10295	High-grade osteosarcoma pre-chemotherapy biopsy L2616int
GSM825657	7.755150022	GPL10295	High-grade osteosarcoma pre-chemotherapy biopsy L2617int
GSM825658	8.290514365	GPL10295	High-grade osteosarcoma pre-chemotherapy biopsy L2618int
GSM825659	7.651782812	GPL10295	High-grade osteosarcoma pre-chemotherapy biopsy L2619int
GSM825660	7.75385913	GPL10295	High-grade osteosarcoma pre-chemotherapy biopsy L2620int
GSM825661	7.452542567	GPL10295	High-grade osteosarcoma pre-chemotherapy biopsy L3431int
GSM825662	7.571275516	GPL10295	High-grade osteosarcoma pre-chemotherapy biopsy L3432int
GSM825663	8.04425807	GPL10295	High-grade osteosarcoma pre-chemotherapy biopsy L3433int
GSM825664	7.593152519	GPL10295	High-grade osteosarcoma pre-chemotherapy biopsy L3434int
GSM825665	7.653432401	GPL10295	High-grade osteosarcoma pre-chemotherapy biopsy L3435int
GSM825666	7.553208301	GPL10295	High-grade osteosarcoma pre-chemotherapy biopsy L3436int
GSM825667	7.639727004	GPL10295	High-grade osteosarcoma pre-chemotherapy biopsy L3437int
GSM825668	7.546437303	GPL10295	High-grade osteosarcoma pre-chemotherapy biopsy L3438int
GSM825669	7.596407796	GPL10295	High-grade osteosarcoma pre-chemotherapy biopsy L3439int
GSM825670	7.719641667	GPL10295	High-grade osteosarcoma pre-chemotherapy biopsy L3440int
GSM825671	8.228352703	GPL10295	High-grade osteosarcoma pre-chemotherapy biopsy L3441int
GSM825672	7.884265029	GPL10295	High-grade osteosarcoma pre-chemotherapy biopsy L3442int
GSM825673	7.455090659	GPL10295	High-grade osteosarcoma pre-chemotherapy biopsy L3443int
GSM825674	7.451993491	GPL10295	High-grade osteosarcoma pre-chemotherapy biopsy L3444int
GSM825675	7.825271907	GPL10295	High-grade osteosarcoma pre-chemotherapy biopsy L3445int
GSM825676	7.694037403	GPL10295	High-grade osteosarcoma pre-chemotherapy biopsy L3446int
GSM825677	7.932547169	GPL10295	High-grade osteosarcoma pre-chemotherapy biopsy L3447int
GSM825678	7.572328421	GPL10295	High-grade osteosarcoma pre-chemotherapy biopsy L3448int
GSM825679	7.775073075	GPL10295	High-grade osteosarcoma pre-chemotherapy biopsy L3449int
GSM825680	7.518057827	GPL10295	High-grade osteosarcoma pre-chemotherapy biopsy L3453int
GSM825681	8.509066314	GPL10295	High-grade osteosarcoma pre-chemotherapy biopsy L3454int
GSM825682	8.384951579	GPL10295	High-grade osteosarcoma pre-chemotherapy biopsy L3455int
GSM825683	7.664854604	GPL10295	High-grade osteosarcoma pre-chemotherapy biopsy L3456int
GSM825684	7.658304401	GPL10295	High-grade osteosarcoma pre-chemotherapy biopsy L3457int
GSM825685	8.199740597	GPL10295	High-grade osteosarcoma pre-chemotherapy biopsy L3458int
GSM825686	8.126999749	GPL10295	High-grade osteosarcoma pre-chemotherapy biopsy L3459int
GSM825687	7.815980625	GPL10295	High-grade osteosarcoma pre-chemotherapy biopsy L3460int
GSM825688	7.75354613	GPL10295	High-grade osteosarcoma pre-chemotherapy biopsy L3461int
GSM825689	7.693431656	GPL10295	High-grade osteosarcoma pre-chemotherapy biopsy L3462int
GSM825690	7.46242631	GPL10295	High-grade osteosarcoma pre-chemotherapy biopsy L3463int
GSM825691	7.524781455	GPL10295	High-grade osteosarcoma pre-chemotherapy biopsy L3464int
GSM825692	7.47429186	GPL10295	High-grade osteosarcoma pre-chemotherapy biopsy L3465int
GSM825693	8.005920303	GPL10295	High-grade osteosarcoma pre-chemotherapy biopsy L3466int
GSM825694	7.541821019	GPL10295	High-grade osteosarcoma pre-chemotherapy biopsy L3467int
GSM825695	7.695990247	GPL10295	High-grade osteosarcoma pre-chemotherapy biopsy L3468int
GSM825696	7.512463187	GPL10295	High-grade osteosarcoma pre-chemotherapy biopsy L3469int
GSM825697	8.394549948	GPL10295	High-grade osteosarcoma pre-chemotherapy biopsy L3470int
GSM825698	7.66405856	GPL10295	High-grade osteosarcoma pre-chemotherapy biopsy L3471int

GSM825699	7.814480334	GPL10295	High-grade osteosarcoma pre-chemotherapy biopsy L3472int
GSM825700	7.790641822	GPL10295	High-grade osteosarcoma pre-chemotherapy biopsy L3473int
GSM825701	8.093862918	GPL10295	High-grade osteosarcoma pre-chemotherapy biopsy L3474int
GSM825702	7.909813219	GPL10295	High-grade osteosarcoma pre-chemotherapy biopsy L3475int
GSM825703	7.708466979	GPL10295	High-grade osteosarcoma pre-chemotherapy biopsy L3476int
GSM825704	7.586175091	GPL10295	High-grade osteosarcoma pre-chemotherapy biopsy L3477int
GSM825705	7.560483648	GPL10295	High-grade osteosarcoma pre-chemotherapy biopsy L3533int
GSM825706	7.55516275	GPL10295	High-grade osteosarcoma pre-chemotherapy biopsy L3534int
GSM825707	7.559983728	GPL10295	High-grade osteosarcoma pre-chemotherapy biopsy L3535int
GSM825708	7.534631221	GPL10295	High-grade osteosarcoma pre-chemotherapy biopsy L3536int
GSM825709	7.526734103	GPL10295	High-grade osteosarcoma pre-chemotherapy biopsy L3538int
GSM879156	7.461736841	GPL16102	Osteosarcoma
GSM879157	7.380462031	GPL16102	Osteosarcoma
GSM879158	7.631817073	GPL16102	Osteosarcoma
GSM879159	8.512594874	GPL16102	Osteosarcoma
GSM879160	8.233593506	GPL16102	Osteosarcoma
GSM879161	7.822594228	GPL16102	Osteosarcoma
GSM879162	7.796264772	GPL16102	Osteosarcoma
GSM879163	7.648956683	GPL16102	Osteosarcoma
GSM879164	8.285188598	GPL16102	Osteosarcoma
GSM879165	7.564918903	GPL16102	Osteosarcoma
GSM879166	7.725018186	GPL16102	Osteosarcoma
GSM879167	7.747628437	GPL16102	Osteosarcoma
GSM879168	8.185049552	GPL16102	Osteosarcoma
GSM879169	7.842014145	GPL16102	Osteosarcoma
GSM879170	7.466894813	GPL16102	Osteosarcoma
GSM879171	7.997723804	GPL16102	Osteosarcoma
GSM879172	7.953425141	GPL16102	Osteosarcoma
GSM879173	7.787077109	GPL16102	Osteosarcoma
GSM879174	7.305457269	GPL16102	Osteosarcoma
GSM879175	8.076220323	GPL16102	Normal osteoblast
GSM879176	7.486351517	GPL16102	Normal osteoblast
GSM879177	7.886140529	GPL16102	Normal bone
GSM879178	7.505849268	GPL16102	Normal bone
GSM879179	7.603822943	GPL16102	Normal bone
GSM879180	7.271502562	GPL16102	Normal bone
GSM958993	7.838963471	GPL96	osteosarcoma
GSM958994	9.981604524	GPL96	osteosarcoma
GSM958995	9.382767187	GPL96	osteosarcoma
GSM958996	10.06760511	GPL96	osteosarcoma
GSM958997	8.323981014	GPL96	osteosarcoma
GSM958998	7.890453846	GPL96	osteosarcoma
GSM958999	7.918329479	GPL96	osteosarcoma
GSM959000	7.479352879	GPL96	osteosarcoma
GSM959001	8.694963716	GPL96	osteosarcoma
GSM959002	8.240978755	GPL96	osteosarcoma

GSM959039	7.740039744	GPL96	undifferentiated osteoblasts grown in proliferation medium
GSM959040	7.958237534	GPL96	differentiated osteoblasts grown in differentiation medium
GSM959043	7.834727986	GPL96	bone marrow-derived mesenchymal stem cells
GSM717846	7.725149434	GPL10295	Mesenchymal stem cell
GSM717847	7.581945571	GPL10295	Mesenchymal stem cell
GSM717848	7.70788472	GPL10295	Mesenchymal stem cell
GSM717849	7.582494941	GPL10295	Mesenchymal stem cell
GSM717850	7.610370658	GPL10295	Mesenchymal stem cell
GSM717851	7.499490509	GPL10295	Mesenchymal stem cell
GSM717852	7.475565305	GPL10295	Mesenchymal stem cell
GSM717853	7.557016603	GPL10295	Mesenchymal stem cell
GSM717854	7.56583806	GPL10295	Mesenchymal stem cell
GSM717855	7.643666737	GPL10295	Mesenchymal stem cell
GSM717856	7.581932934	GPL10295	Mesenchymal stem cell
GSM717857	7.569726137	GPL10295	Mesenchymal stem cell
GSM825623	8.035499018	GPL10295	Osteoblast
GSM825624	7.895234483	GPL10295	Osteoblast
GSM825625	7.754401898	GPL10295	Osteoblast
GSM825626	7.983967697	GPL10295	High-grade osteosarcoma pre-chemotherapy biopsy/tibia/fibulaF
GSM825627	7.704090315	GPL10295	High-grade osteosarcoma pre-chemotherapy biopsy/femur/F
GSM825628	7.51312935	GPL10295	High-grade osteosarcoma pre-chemotherapy biopsy/tibia/fibula/F
GSM825629	8.107275821	GPL10295	High-grade osteosarcoma pre-chemotherapy biopsy/femur/F
GSM825630	7.670882398	GPL10295	High-grade osteosarcoma pre-chemotherapy biopsy/tibia/fibula/M
GSM825631	7.553985172	GPL10295	High-grade osteosarcoma pre-chemotherapy biopsy/tibia/fibula/M
GSM825632	7.574400577	GPL10295	High-grade osteosarcoma pre-chemotherapy biopsy/tibia/fibula/M
GSM825633	7.661105847	GPL10295	High-grade osteosarcoma pre-chemotherapy biopsy/femur/M
GSM825634	7.688257221	GPL10295	High-grade osteosarcoma pre-chemotherapy biopsy/femur/M
GSM825635	7.496317287	GPL10295	High-grade osteosarcoma pre-chemotherapy biopsy/femur/M
GSM825636	7.673973769	GPL10295	High-grade osteosarcoma pre-chemotherapy biopsy/femur/M
GSM825637	7.797207625	GPL10295	High-grade osteosarcoma pre-chemotherapy biopsy/tibia/fibula/F
GSM825638	7.770380208	GPL10295	High-grade osteosarcoma pre-chemotherapy biopsy/femur/F
GSM825639	7.640796799	GPL10295	High-grade osteosarcoma pre-chemotherapy biopsy/tibia/fibula/M
GSM825640	7.543910411	GPL10295	High-grade osteosarcoma pre-chemotherapy biopsy/humerus/M
GSM825641	7.821689727	GPL10295	High-grade osteosarcoma pre-chemotherapy biopsy/femur/M
GSM825642	7.636564422	GPL10295	High-grade osteosarcoma pre-chemotherapy biopsy/femur/M
GSM825643	7.727378325	GPL10295	High-grade osteosarcoma pre-chemotherapy biopsy/femur/F
GSM825644	7.728246934	GPL10295	High-grade osteosarcoma pre-chemotherapy biopsy/tibia/fibula/M
GSM825645	7.444334435	GPL10295	High-grade osteosarcoma pre-chemotherapy biopsy/femur/F
GSM825646	7.452363696	GPL10295	High-grade osteosarcoma pre-chemotherapy biopsy/tibia/fibula/M
GSM825647	7.776465692	GPL10295	High-grade osteosarcoma pre-chemotherapy biopsy/tibia/fibula/F
GSM825648	7.497378261	GPL10295	High-grade osteosarcoma pre-chemotherapy biopsy/tibia/fibula/M
GSM825649	7.646537053	GPL10295	High-grade osteosarcoma pre-chemotherapy biopsy/humerus/F
GSM825650	7.435562819	GPL10295	High-grade osteosarcoma pre-chemotherapy biopsy/femur/F
GSM825651	7.709310064	GPL10295	High-grade osteosarcoma pre-chemotherapy biopsy/humerus/F
GSM825652	7.481747729	GPL10295	High-grade osteosarcoma pre-chemotherapy biopsy/femur/F
GSM825653	7.749049819	GPL10295	High-grade osteosarcoma pre-chemotherapy biopsy/tibia/fibula/M

GSM825654	7.436044038	GPL10295	High-grade osteosarcoma pre-chemotherapy biopsy/femur/M
GSM825655	7.51551564	GPL10295	High-grade osteosarcoma pre-chemotherapy biopsy/tibia/fibula/M
GSM825656	7.474996	GPL10295	High-grade osteosarcoma pre-chemotherapy biopsy/tibia/fibula/M
GSM825657	7.755150022	GPL10295	High-grade osteosarcoma pre-chemotherapy biopsy/tibia/fibula/F
GSM825658	8.290514365	GPL10295	High-grade osteosarcoma pre-chemotherapy biopsy/femur/M
GSM825659	7.651782812	GPL10295	High-grade osteosarcoma pre-chemotherapy biopsy/femur/F
GSM825660	7.75385913	GPL10295	High-grade osteosarcoma pre-chemotherapy biopsy/femur/M
GSM825661	7.452542567	GPL10295	High-grade osteosarcoma pre-chemotherapy biopsy/humerus/F
GSM825662	7.571275516	GPL10295	High-grade osteosarcoma pre-chemotherapy biopsy/tibia/fibula/M
GSM825663	8.04425807	GPL10295	High-grade osteosarcoma pre-chemotherapy biopsy/femur/F
GSM825664	7.593152519	GPL10295	High-grade osteosarcoma pre-chemotherapy biopsy/tibia/fibula/M
GSM825665	7.653432401	GPL10295	High-grade osteosarcoma pre-chemotherapy biopsy/femur/M
GSM825666	7.553208301	GPL10295	High-grade osteosarcoma pre-chemotherapy biopsy/femur/M
GSM825667	7.639727004	GPL10295	High-grade osteosarcoma pre-chemotherapy biopsy/femur/M
GSM825668	7.546437303	GPL10295	High-grade osteosarcoma pre-chemotherapy biopsy/tibia/fibula/M
GSM825669	7.596407796	GPL10295	High-grade osteosarcoma pre-chemotherapy biopsy/humerus/M
GSM825670	7.719641667	GPL10295	High-grade osteosarcoma pre-chemotherapy biopsy/femur/M
GSM825671	8.228352703	GPL10295	High-grade osteosarcoma pre-chemotherapy biopsy/femur/M
GSM825672	7.884265029	GPL10295	High-grade osteosarcoma pre-chemotherapy biopsy/femur/F
GSM825673	7.455090659	GPL10295	High-grade osteosarcoma pre-chemotherapy biopsy/humerus/F
GSM825674	7.451993491	GPL10295	High-grade osteosarcoma pre-chemotherapy biopsy/humerus/M
GSM825675	7.825271907	GPL10295	High-grade osteosarcoma pre-chemotherapy biopsy/tibia/fibula/F
GSM825676	7.694037403	GPL10295	High-grade osteosarcoma pre-chemotherapy biopsy/femur/M
GSM825677	7.932547169	GPL10295	High-grade osteosarcoma pre-chemotherapy biopsy/humerus/M
GSM825678	7.572328421	GPL10295	High-grade osteosarcoma pre-chemotherapy biopsy/femur/M
GSM825679	7.775073075	GPL10295	High-grade osteosarcoma pre-chemotherapy biopsy/femur/F
GSM825680	7.518057827	GPL10295	High-grade osteosarcoma pre-chemotherapy biopsy/femur/M
GSM825681	8.509066314	GPL10295	High-grade osteosarcoma pre-chemotherapy biopsy/other/M
GSM825682	8.384951579	GPL10295	High-grade osteosarcoma pre-chemotherapy biopsy/tibia/fibula/M
GSM825683	7.664854604	GPL10295	High-grade osteosarcoma pre-chemotherapy biopsy/tibia/fibula/M
GSM825684	7.658304401	GPL10295	High-grade osteosarcoma pre-chemotherapy biopsy/humerus/F
GSM825685	8.199740597	GPL10295	High-grade osteosarcoma pre-chemotherapy biopsy/femur/M
GSM825686	8.126999749	GPL10295	High-grade osteosarcoma pre-chemotherapy biopsy/tibia/fibula/F
GSM825687	7.815980625	GPL10295	High-grade osteosarcoma pre-chemotherapy biopsy/tibia/fibula/M
GSM825688	7.75354613	GPL10295	High-grade osteosarcoma pre-chemotherapy biopsy/femur/M
GSM825689	7.693431656	GPL10295	High-grade osteosarcoma pre-chemotherapy biopsy/tibia/fibula/M
GSM825690	7.46242631	GPL10295	High-grade osteosarcoma pre-chemotherapy biopsy/humerus/M
GSM825691	7.524781455	GPL10295	High-grade osteosarcoma pre-chemotherapy biopsy/tibia/fibula/M
GSM825692	7.47429186	GPL10295	High-grade osteosarcoma pre-chemotherapy biopsy/femur/F
GSM825693	8.005920303	GPL10295	High-grade osteosarcoma pre-chemotherapy biopsy/femur/F
GSM825694	7.541821019	GPL10295	High-grade osteosarcoma pre-chemotherapy biopsy/femur/M
GSM825695	7.695990247	GPL10295	High-grade osteosarcoma pre-chemotherapy biopsy/other/M
GSM825696	7.512463187	GPL10295	High-grade osteosarcoma pre-chemotherapy biopsy/femur/M
GSM825697	8.394549948	GPL10295	High-grade osteosarcoma pre-chemotherapy biopsy/unknown/F
GSM825698	7.66405856	GPL10295	High-grade osteosarcoma pre-chemotherapy biopsy/axial/F
GSM825699	7.814480334	GPL10295	High-grade osteosarcoma pre-chemotherapy biopsy/femur/M

GSM825700	7.790641822	GPL10295	High-grade osteosarcoma pre-chemotherapy biopsy/tibia/fibula/M
GSM825701	8.093862918	GPL10295	High-grade osteosarcoma pre-chemotherapy biopsy/humerus/M
GSM825702	7.909813219	GPL10295	High-grade osteosarcoma pre-chemotherapy biopsy/femur/M
GSM825703	7.708466979	GPL10295	High-grade osteosarcoma pre-chemotherapy biopsy/tibia/fibula/M
GSM825704	7.586175091	GPL10295	High-grade osteosarcoma pre-chemotherapy biopsy/femur/M
GSM825705	7.560483648	GPL10295	High-grade osteosarcoma pre-chemotherapy biopsy/femur/M
GSM825706	7.55516275	GPL10295	High-grade osteosarcoma pre-chemotherapy biopsy/femur/F
GSM825707	7.559983728	GPL10295	High-grade osteosarcoma pre-chemotherapy biopsy/tibia/fibula/M
GSM825708	7.534631221	GPL10295	High-grade osteosarcoma pre-chemotherapy biopsy/femur/M
GSM825709	7.526734103	GPL10295	High-grade osteosarcoma pre-chemotherapy biopsy
GSM1038236	7.56699047	GPL10295	High-grade osteosarcoma cell line
GSM1038237	7.697653745	GPL10295	High-grade osteosarcoma cell line
GSM1038238	7.806908137	GPL10295	High-grade osteosarcoma cell line
GSM1038239	8.256594883	GPL10295	High-grade osteosarcoma cell line
GSM1038240	8.232304927	GPL10295	High-grade osteosarcoma cell line
GSM1038241	7.869700887	GPL10295	High-grade osteosarcoma cell line
GSM1038242	7.838225185	GPL10295	High-grade osteosarcoma cell line
GSM1038243	7.722003822	GPL10295	High-grade osteosarcoma cell line
GSM1038244	8.471365154	GPL10295	High-grade osteosarcoma cell line
GSM1038245	7.64943764	GPL10295	High-grade osteosarcoma cell line
GSM1038246	7.795922097	GPL10295	High-grade osteosarcoma cell line
GSM1038247	8.184228145	GPL10295	High-grade osteosarcoma cell line
GSM1038248	7.886640446	GPL10295	High-grade osteosarcoma cell line
GSM1038249	7.573491856	GPL10295	High-grade osteosarcoma cell line
GSM1038250	7.50279617	GPL10295	High-grade osteosarcoma cell line
GSM1038251	8.00770881	GPL10295	High-grade osteosarcoma cell line
GSM1038252	7.98214656	GPL10295	High-grade osteosarcoma cell line
GSM1038253	7.837193609	GPL10295	High-grade osteosarcoma cell line
GSM1038254	7.44188723	GPL10295	High-grade osteosarcoma cell line
GSM1192008	7.363715	GPL6947	1:METASTATIC-yes
GSM1192009	7.24369	GPL6947	2:METASTATIC-yes
GSM1192010	7.33975	GPL6947	3:METASTATIC-yes
GSM1192011	7.329625	GPL6947	4:METASTATIC-yes
GSM1192012	7.304785	GPL6947	5:METASTATIC-yes
GSM1192013	7.31283	GPL6947	6:METASTATIC-yes
GSM1192014	7.314025	GPL6947	7:METASTATIC-no
GSM1192015	7.3297	GPL6947	8:METASTATIC-no
GSM1192016	7.36553	GPL6947	9:METASTATIC-no
GSM1192017	7.2846	GPL6947	10:METASTATIC-no
GSM1192018	7.32781	GPL6947	11:METASTATIC-no
GSM1192019	7.320385	GPL6947	12:METASTATIC-no
GSM1676300	8.8616194	GPL11028	Osteosarcoma
GSM1676303	8.016198492	GPL11028	Osteosarcoma
GSM1676308	5.671001444	GPL11028	Osteosarcoma
GSM1676314	7.900893518	GPL11028	Osteosarcoma
GSM1676316	8.475353647	GPL11028	Osteosarcoma

GSM1676324	7.62162095	GPL11028	Osteosarcoma
GSM1676341	7.736751456	GPL11028	Osteosarcoma
GSM1676345	8.500601764	GPL11028	Normal human mesenchymal stem cell
GSM1676346	6.861644672	GPL11028	Normal human chondrocyte
GSM1676347	7.516560519	GPL11028	Normal human osteoblast
GSM1676348	8.73656388	GPL11028	Normal human skeletal muscle
GSM1676349	6.932180802	GPL11028	Osteosarcoma
GSM1676352	8.328760212	GPL11028	Osteosarcoma
GSM1676359	8.543522364	GPL11028	Osteosarcoma
GSM2276632	9.2443285	GPL570	Bone primary cells
GSM2276633	9.225092	GPL570	Bone primary cells
GSM2276634	8.155055	GPL570	Bone primary cells
GSM2276635	5.8218265	GPL570	Lung metastasis cells
GSM2276636	6.988139	GPL570	Lung metastasis cells
GSM2276637	8.191608	GPL570	Lung metastasis cells
GSM2335686	33.9608	GPL11154	tumor type: metastasis
GSM2335687	6.68349	GPL11154	tumor type: primary
GSM2335688	12.5737	GPL11154	tumor type: primary
GSM2335689	17.2254	GPL11154	tumor type: primary
GSM2335690	2.71915	GPL11154	tumor type: primary
GSM2335691	5.4131	GPL11154	tumor type: primary
GSM2335692	4.08658	GPL11154	tumor type: primary
GSM2335693	10.271	GPL11154	tumor type: primary
GSM2335694	6.33505	GPL11154	tumor type: primary
GSM2335695	1.47133	GPL11154	tumor type: primary
GSM2335696	7.59695	GPL11154	tumor type: primary
GSM2335700	11.0041	GPL11154	tumor type: primary
GSM2335701	10.4041	GPL11154	tumor type: primary
GSM2335702	11.5934	GPL11154	tumor type: primary
GSM2335703	6.34012	GPL11154	tumor type: primary
GSM2335704	5.19214	GPL11154	tumor type: primary
GSM2335705	7.24554	GPL11154	tumor type: primary
GSM2335706	2.74129	GPL11154	tumor type: primary
GSM2335707	3.65726	GPL11154	tumor type: primary
GSM2335712	2.53269	GPL11154	tumor type: metastasis
GSM2335713	3.68722	GPL11154	tumor type: metastasis
GSM2335714	23.1899	GPL11154	tumor type: primary
GSM2335715	14.307	GPL11154	tumor type: metastasis
GSM2335716	11.7376	GPL11154	tumor type: metastasis
GSM2335717	21.1156	GPL11154	tumor type: metastasis
GSM2335718	6.33892	GPL11154	tumor type: metastasis
GSM2335719	30.6418	GPL11154	tumor type: metastasis
GSM2335720	12.8956	GPL11154	tumor type: primary
GSM2335721	10.9024	GPL11154	tumor type: metastasis
GSM2335722	18.3368	GPL11154	tumor type: primary
GSM2335723	7.25944	GPL11154	tumor type: primary

GSM2335724	4.39789	GPL11154	tumor type: primary
GSM2335725	4.95524	GPL11154	tumor type: primary
GSM2335730	7.97358	GPL11154	tumor type: normal bone
GSM2335731	5.91784	GPL11154	tumor type: normal bone
GSM2335732	0.195058	GPL11154	tumor type: normal bone
GSM3593746	6.657607037	GPL20301	tissue: tumor/gender: female/age: 15
GSM3593747	5.969065562	GPL20301	tissue: tumor/gender: male/age: 46
GSM3593748	5.721689388	GPL20301	tissue: adjacent normal/gender: male/age: 46
GSM3593750	4.790231634	GPL20301	tissue: adjacent normal/gender: male/age: 22
GSM3593752	4.199197701	GPL20301	tissue: adjacent normal/gender: male/age: 10
GSM3593754	1.684204459	GPL20301	tissue: adjacent normal/gender: female/age: 10
GSM4272756	5.234217331	GPL20301	tissue: normal/gender: male/age: 53
GSM4272757	4.187475304	GPL20301	tissue: tumor/gender: male/age: 53
GSM4272758	6.659889498	GPL20301	tissue: normal/gender: male/age: 39
GSM4272759	4.013581391	GPL20301	tissue: tumor/gender: male/age: 39
GSM4272760	6.112390755	GPL20301	tissue: normal/gender: male/age: 15
GSM4272762	5.325062893	GPL20301	tissue: normal/gender: male/age: 15
GSM4955769	3.333445923	GPL20301	tissue: normal/gender: female/age: 5
GSM4955771	3.072885541	GPL20301	tissue: normal/gender: male/age: 45
TARGET.40.PASUUH.01A	0.861637817	None	Non-metastatic (confirmed)
TARGET.40.PAUTWB.01A	2.654298047	None	Non-metastatic (confirmed)
TARGET.40.PAKUZU.01A	1.841127778	None	Non-metastatic (confirmed)
TARGET.40.PARJXU.01A	4.022465469	None	Metastatic (confirmed)
TARGET.40.PAPWWC.01A	1.507211103	None	Non-metastatic (confirmed)
TARGET.40.PAUUML.01A	0.686522938	None	Non-metastatic (confirmed)
TARGET.40.PAMHLF.01A	2.31089266	None	Non-metastatic (confirmed)
TARGET.40.PAUBIT.01A	3.118525849	None	Non-metastatic (confirmed)
TARGET.40.PASFCV.01A	1.256889886	None	Non-metastatic (confirmed)
TARGET.40.PARDAX.01A	0.473838853	None	Metastatic (confirmed)
TARGET.40.0A4I4O.01A	2.243364426	None	Non-metastatic (confirmed)
TARGET.40.0A4I48.01A	2.59724511	None	Metastatic
TARGET.40.PASEFS.01A	3.949039943	None	Non-metastatic (confirmed)
TARGET.40.PARKAF.01A	1.716595882	None	Non-metastatic (confirmed)
TARGET.40.PATUXZ.01A	1.468270504	None	Metastatic (confirmed)
TARGET.40.PAMHYN.01A	3.030177144	None	Non-metastatic (confirmed)
TARGET.40.PASEBY.01A	2.703676599	None	Non-metastatic (confirmed)
TARGET.40.PANMIG.01A	3.473046545	None	Metastatic
TARGET.40.PAKFVX.01A	0.60757888	None	Non-metastatic (confirmed)
TARGET.40.PASNZV.01A	0.409255147	None	Non-metastatic (confirmed)
TARGET.40.0A4I4M.01A	1.273873908	None	Non-metastatic (confirmed)
TARGET.40.PAMYYJ.01A	0.544386466	None	Metastatic
TARGET.40.PAVECB.01A	3.333423734	None	Non-metastatic (confirmed)
TARGET.40.0A4I42.01A	1.492673596	None	Non-metastatic (confirmed)
TARGET.40.PASYUK.01A	1.999386724	None	Non-metastatic (confirmed)
TARGET.40.PALECC.01A	2.215740713	None	Non-metastatic (confirmed)
TARGET.40.PAMLKS.01A	0.214497868	None	Metastatic

TARGET.40.PANSEN.01A	3.379080266	None	Non-metastatic (confirmed)
TARGET.40.PALHRL.01A	2.411995618	None	Metastatic
TARGET.40.PANZHX.01A	1.329869872	None	Metastatic
TARGET.40.PALKDP.01A	2.074197135	None	Non-metastatic (confirmed)
TARGET.40.0A4I65.01A	1.854235079	None	Non-metastatic (Confirmed)
TARGET.40.0A4I6O.01A	3.415014957	None	Non-metastatic (confirmed)
TARGET.40.PALFYN.01A	2.192572941	None	Non-metastatic (confirmed)
TARGET.40.PATPBS.01A	1.47990311	None	Non-metastatic (confirmed)
TARGET.40.PAPXGT.01A	3.222989986	None	Non-metastatic (confirmed)
TARGET.40.0A4I4E.01A	2.077687363	None	Metastatic
TARGET.40.0A4HLD.01A	3.687799768	None	Non-metastatic (confirmed)
TARGET.40.PAUYTT.01A	3.002990491	None	Non-metastatic (confirmed)
TARGET.40.0A4I0W.01A	1.927934369	None	Non-metastatic (confirmed)
TARGET.40.PARFTG.01A	1.930964367	None	Non-metastatic (confirmed)
TARGET.40.0A4HX8.01A	3.374037888	None	Non-metastatic (confirmed)
TARGET.40.PAUTYB.01A	1.185866545	None	Non-metastatic (confirmed)
TARGET.40.PATMXR.01A	1.640343282	None	Metastatic
TARGET.40.PAPKWD.01A	1.47134356	None	Non-metastatic (confirmed)
TARGET.40.PAPNVD.01A	2.719424417	None	Non-metastatic (confirmed)
TARGET.40.PATMPU.01A	2.468531186	None	Non-metastatic (confirmed)
TARGET.40.PAPFLB.01A	2.668799909	None	Non-metastatic (confirmed)
TARGET.40.PAPIJR.01A	2.165526097	None	Non-metastatic (confirmed)
TARGET.40.PAMEKS.01A	1.447684981	None	Metastatic
TARGET.40.PAVDTY.01A	0.53216732	None	Non-metastatic (confirmed)
TARGET.40.PANGPE.01A	2.884832328	None	Non-metastatic (confirmed)
TARGET.40.PAMJXS.01A	2.31533436	None	Non-metastatic (confirmed)
TARGET.40.PAKZZK.01A	3.208080202	None	Non-metastatic (confirmed)
TARGET.40.PALWWX.01A	1.064537998	None	Metastatic
TARGET.40.PASKZZ.01A	2.426452628	None	Non-metastatic (confirmed)
TARGET.40.0A4HXS.01A	2.562523303	None	Non-metastatic (confirmed)
TARGET.40.PATEEM.01A	4.509809834	None	Non-metastatic (confirmed)
TARGET.40.0A4I0Q.01A	2.658645654	None	Metastatic
TARGET.40.PALKGN.01A	2.118193584	None	Non-metastatic (confirmed)
TARGET.40.PASRNE.01A	0.392207499	None	Non-metastatic (confirmed)
TARGET.40.PARGTM.01A	3.393210243	None	Non-metastatic (confirmed)
TARGET.40.PAUVUL.01A	1.088548697	None	Metastatic (confirmed)
TARGET.40.PANGRW.01A	1.050188223	None	Non-metastatic (confirmed)
TARGET.40.PAVCLP.01A	2.895263839	None	Non-metastatic (confirmed)
TARGET.40.PATAWV.01A	0.843983844	None	Non-metastatic (confirmed)
TARGET.40.PARBGW.01A	1.422448312	None	Metastatic (confirmed)
TARGET.40.PANXSC.01A	2.408983529	None	Non-metastatic (confirmed)
TARGET.40.0A4HMC.01A	3.435428576	None	Non-metastatic (confirmed)
TARGET.40.PAUXPZ.01A	2.307632528	None	Metastatic (confirmed)
TARGET.40.PATJVI.01A	0.027012465	None	Metastatic (confirmed)
TARGET.40.PAMRHD.01A	2.766722359	None	Metastatic
TARGET.40.PALZGU.01A	2.365384595	None	Non-metastatic (confirmed)

TARGET.40.PANZZJ.01A	2.037206487	None	Non-metastatic (confirmed)
TARGET.40.PATKSS.01A	3.543607236	None	Non-metastatic (confirmed)
TARGET.40.PATMIF.01A	2.63501138	None	Non-metastatic (confirmed)
TARGET.40.PANPUM.01A	3.136174977	None	Metastatic
TARGET.40.PAKXLD.01A	3.653862565	None	Non-metastatic (confirmed)
TARGET.40.PASSLM.01A	1.899252985	None	Metastatic (confirmed)
TARGET.40.PAMTCM.01A	1.848117126	None	Non-metastatic (confirmed)
TARGET.40.0A4HY5.01A	1.677846466	None	Metastatic
TARGET.40.PAVALD.01A	2.646508309	None	Non-metastatic (confirmed)
TARGET.40.0A4I8U.01A	2.925600724	None	Non-metastatic (confirmed)
TARGET.40.0A4I9K.01A	2.942064033	None	Non-metastatic (Confirmed)
TARGET.40.0A4I3S.01A	0.852078763	None	Non-metastatic (confirmed)
TARGET.40.PANVJJ.01A	2.001730196	None	Non-metastatic (confirmed)
TARGET.40.0A4I5B.01A	2.667051917	None	Non-metastatic (confirmed)

Supplementary table2: the 2D structure and PK/PD parameters of Exisulind.

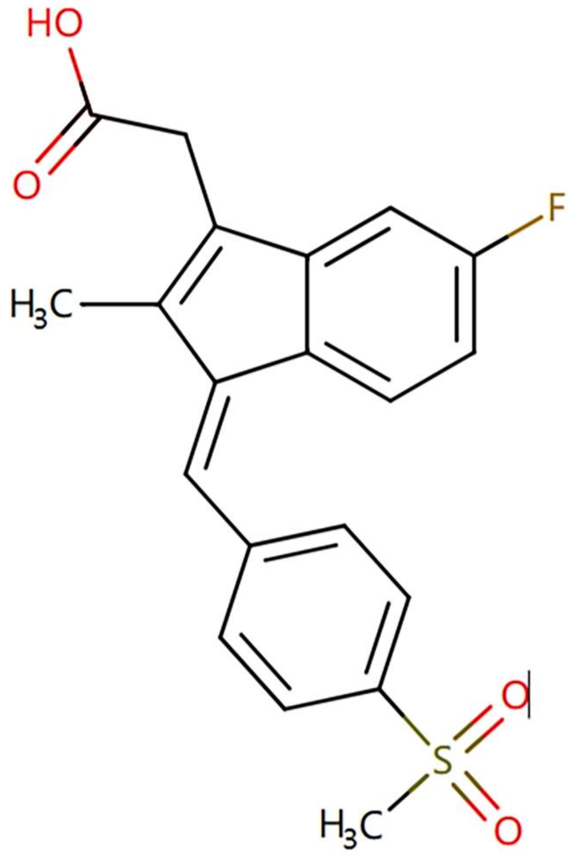

Exisulind:

Pharmacokinetics:

Orally administered doses from 50 to 400 mg of Exisulind are rapidly absorbed in norma healthy volunteers The maximum concentration (C_{max}), time to C_{max} (T_{max}), and area under the concentration curve (AUC) are 10.7 ug/ml, 2.3 h and 125 ug/h/ml, respectively for patients receiving 200 mg twice-daily. The half-life is 9-14 h. The plasma concentrations showed multiple peaks suggesting enterohepatic recirculation. The T_{max} and C_{max} are increased when the medication was taken with food; however, the AUCs are bioequivalent in the fed and fasting states. Exisulind is largely excreted unchanged in the bile and appears to exert no effect on the cytochrome P450 enzymes [1].

Pharmacodynamics:

Exisulind has been shown to inhibit growth and induce apoptosis in several types of solid tumor cells including colon, prostate, bladder, breast, lung, cervical and hepatic with minimal or no effect on normal cells. In addition, Exisulind and its analogues inhibited leukemia and myeloma cell lines. In terms of mechanism of action, Exisulind inhibits cGMP PDE thereby increasing cellular levels of cGMP and activating protein kinase G (PKG). In proliferating HT-29 cells, Exisulind blocked G1 cell cycle progression but also induced apoptosis. Exisulind did not affect cell cycle progression in non-proliferating cells but did inhibit cell growth and induced apoptosis. Taken together, Exisulind causes growth inhibition and apoptosis under conditions where cell cycle arrest does not occur [2].

1. Webster WS, Leibovich BC. Exisulind in the treatment of prostate cancer. Expert Rev Anticancer Ther. 2005; 5: 957-62.

2. Goluboff ET. Exisulind, a selective apoptotic antineoplastic drug. Expert Opin Investig Drugs. 2001; 10: 1875-82.

Supplementary table 3 : the 2D structure and PK/PD parameters of Tacrolimus.

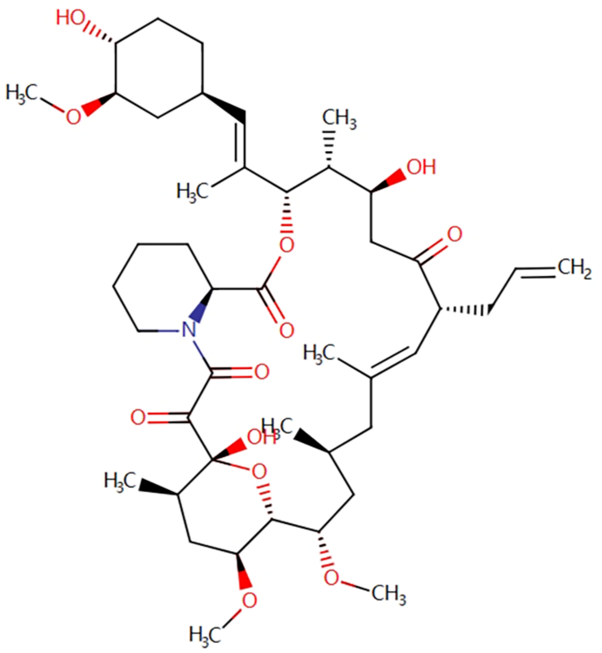

Tacrolimus (FK-506):

Pharmacokinetics:

The pharmacokinetics of FK-506 are variable among patients, and the absorption of the drug after oral administration is variable and incomplete. This indicates the need for monitoring the plasma concentrations of FK-506 in patients to avoid potential rejection of the transplanted organ. Since the distribution of FK-506 in blood is temperature-dependent, plasma must be separated at carefully controlled temperatures. In the preliminary observations, the trough concentrations of FK-506 range from <0.1 ng/ml to nearly 5 ng/ml after an oral dose of 0.15 mg/kg/d. There is no specific side effect associated with higher trough concentrations [1].

Pharmacodynamics:

Tacrolimus acts by reducing peptidyl-prolyl isomerase activity by binding to the immunophilin FKBP-12 (FK506 binding protein) creating a new complex. This inhibits both T-lymphocyte signal transduction and IL-2 transcription. Tacrolimus has similar activity to cyclosporine but rates of rejection are lower with tacrolimus. Tacrolimus has also been shown to be effective in the topical treatment of eczema, particularly atopic eczema. It suppresses inflammation in a similar way to steroids, but is not as powerful. An important dermatological advantage of tacrolimus is that it can be used directly on the face; topical steroids cannot be used on the face, as they thin the skin dramatically there. On other parts of the body, topical steroid are generally a better treatment [https://go.drugbank.com/drugs/DB00864].

1. Venkataramanan R, Jain A, Cadoff E, Warty V, Iwasaki K, Nagase K, et al. Pharmacokinetics of FK 506: preclinical and clinical studies. Transplant Proc. 1990; 22: 52-6.
